# A Combined‐Mode Machine Learning Model for Predicting Stroke Recurrence During Hospitalization in Patients with Acute Minor Ischemic Stroke

**DOI:** 10.1002/mco2.70234

**Published:** 2025-05-19

**Authors:** Wanxing Ye, Jin Gan, Meng Wang, Ziyang Liu, Hongqiu Gu, Xin Yang, Chunjuan Wang, Xia Meng, Yong Jiang, Hao Li, Liping Liu, Yongjun Wang, Zixiao Li

**Affiliations:** ^1^ China National Clinical Research Center for Neurological Diseases Beijing Tiantan Hospital Capital Medical University Beijing China; ^2^ Beijing Tiantan Hospital Capital Medical University Beijing China; ^3^ National Center for Healthcare Quality Management in Neurological Diseases Beijing Tiantan Hospital Capital Medical University Beijing China; ^4^ Beijing Advanced Innovation Center for Biomedical Engineering School of Biological Science and Medical Engineering Beihang University Beijing China; ^5^ Advanced Innovation Center for Human Brain Protection Capital Medical University Beijing China; ^6^ Research Unit of Artificial Intelligence in Cerebrovascular Disease Chinese Academy of Medical Sciences Beijing China; ^7^ Center for Excellence in Brain Science and Intelligence Technology Chinese Academy of Sciences Shanghai China

**Keywords:** acute minor ischemic stroke, in‐hospital recurrence, predictive model, machine learning

## Abstract

Acute minor ischemic stroke patients often experience recurrence shortly after symptom onset, highlighting the importance of predicting stroke recurrence for guiding treatment decisions. This study evaluated the effectiveness of machine learning models in predicting in‐hospital recurrence. The study cohort comprised 322,135 patients with acute minor ischemic stroke from 1439 centers, as established by Chinese Stroke Center Alliance. Patients were randomly allocated into training and test sets by different centers. Models including extreme gradient boosting (XGB), light gradient boosting (LGB), and adaptive boosting (ADA) were developed using fivefold cross‐validation on the training set. Optimization was performed for all models based on the most important variable, history of ischemic stroke. Compared with the traditional generalized linear model (GLM), the XGB, LGB, ADA models yielded area under the curve (AUC) values ranging from 0.788 to 0.803 after optimization. All models showed significant improvements in AUC compared with GLM, with LGB exhibiting the most substantial enhancement after optimization. For the first time, this study developed models specifically designed to predict in‐hospital stroke recurrence in acute minor ischemic stroke patients. This finding aids in identifying high‐risk patients and prompts physicians to provide targeted treatment. However, further external validation is warranted to confirm the model's generalizability.

## Graphical abstract

Study evaluated machine learning methods for predicting in‐hospital stroke recurrence in minor ischemic stroke patients. A cohort comprised 322,135 patients from 1439 centers established by Chinese Stroke Center Alliance was used to develop and test models. Compared with the traditional generalized linear model, light gradient boosting model exhibited the most substantial improvement in AUC. This finding helps identifying high‐risk stroke patients.

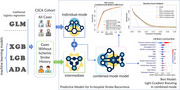

## 1 Introduction

Stroke is a leading causes of morbidity and mortality globally [1, 2, 3]. According to the 2019 Global Burden of Disease Study, ischemic stroke accounts for 62.4% of all stroke events [4]. In China, the lifetime risk of stroke for Chinese residents is as high as 39.3%, making it one of the countries with the highest lifetime stroke risk [5]. This has imposed a heavy burden on the healthcare system.

Ischemic stroke is the most common type of stroke, characterized by unstable prognosis, susceptibility to recurrence, and an elevated risk of early recurrence [6]. In ischemic stroke, minor strokes account for a high proportion, with a rate of over 51.7% [7, 8]. Previous stroke studies have shown that stroke recurrence is more likely occur in the early period after onset [5, 6, 9], with the recurrence rate following a pattern that the shorter the time since stroke onset, the higher the recurrence rate. Lin et al.’s [10] systematic review and meta‐analysis showed that the recurrence rate within the first 3 months after the initial stroke was 7.7%, with a cumulative recurrence rate of 9.5% at 6 months. This indicates that the recurrence rate from 3 to 6 months was 1.8%, which was significantly lower than that within the first 3 months. Moreover, compared with patients with a single episode of ischemic stroke, stroke recurrence leads to more adverse outcomes and has a more severe impact on the patient's quality of life [11]. Patients who adhere to guideline‐based secondary stroke prevention have a lower rate of new strokes [12]. The CHANCE study demonstrated that early dual antiplatelet therapy with the combination of clopidogrel and aspirin within 24 h after the onset of symptoms reduced the recurrence rate from 11.7 to 8.2% within 3 months in minor ischemic stroke and transient ischemic attacks (TIA) patients [13]. This highlights the importance of early prevention in stroke and has attracted considerable attention.

Utilizing predictive models to accurately predict the risk of new‐onset stroke [14], forecast early stroke recurrence, precisely stratify risk in stroke patients, manage intervention targets related to recurrence [15, 16], and further reduce early recurrence are essential aspects of secondary stroke prevention [17, 18]. In recent years, stroke researchers have indeed been committed to developing clinical scores or risk models for predicting stroke recurrence [19]. These predictive models are each specifically applicable to ischemic stroke patients, TIA patients, and mixed populations [15, 20]. Among these, there are the Fukuoka Stroke Risk Score for Japanese (FSRJ) [21], the Essen Stroke Risk Score (ESRS) [22, 23], and the Recurrence Risk Estimator at 90 days (RRE‐90) [24] for ischemic stroke patients, the Stroke Prognosis Instrument‐I (SPI‐I) [25] and its upgraded version SPI‐II [26] for suspected carotid TIA or minor stroke populations, the ABCD^2^‐MRI score [27] in ABCD (age, blood pressure, clinical features, diabetes mellitus) series [28], the Dutch TIA scoring system [29], and the Life Long After Cerebral Ischemia (LiLAC) scoring system [30] for mixed populations of TIA and minor stroke. However, most of these stroke recurrence risk prediction models focus on predicting recurrence over 1 year or even longer [31], except for the RRE‐90 and ABCD^2^‐MRI, which predict recurrence within 90 days. The recurrence period addressed by these models is much longer than the hospital stays for most patients. Therefore, there has been a lack of studies addressing in‐hospital recurrence of minor ischemic strokes.

To address this, our study utilized the multicenter‐registered China Stoke Center Alliance (CSCA) database to establish machine learning‐based prediction models for in‐hospital stroke recurrence in patients with acute minor ischemic stroke, using the R language. We compared the predictive performance of machine learning models with traditional logistic regression models, aiming to provide a reference for developing a comprehensive in‐hospital recurrence prediction model for patients with minor ischemic stroke.

## 2 Results

### 2.1 Baseline Characteristics of Included Patients and Dataset Division

**FIGURE 1:**
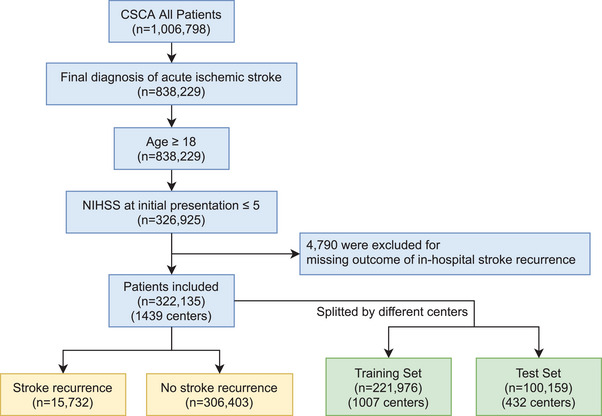
Study inclusion and exclusion flowchart. *Abbreviations*: CSCA, Chinese stroke center alliance; NIHSS, National Institutes of Health Stroke Scale. Created using draw.io‐desktop version 14.6.13.

In this study, 322,135 patients from 1439 centers with acute minor ischemic stroke were included (Figure 1), with a mean age of 65.1 ± 11.9 years, and 208,011 (64.6%) were male. Among these patients, 15,732 (4.88%) experienced stroke recurrence during hospitalization over a median length of stay of 11 days. The baseline characteristics of patients with and without stroke recurrence were shown in Table S1. Various variables, including medical histories, medication histories, and examinations after admission, exhibited statistically significant differences between the recurrence and nonrecurrence groups. However, to assess the predictive power of each variable accurately under large datasets, multivariate logistic regression (MLR) was employed subsequently. The included patients were randomly divided into a training set of 221,976 cases from 1007 centers and a test set of 100,159 cases from 432 centers (Figure 1). The in‐hospital stroke recurrence rates in the two groups were 4.90% (10,883 out of 221,976) and 4.84% (4849 out of 100,159), respectively, with no statistically significant difference (*χ* ^2 ^= 0.548, *p* = 0.459).

### 2.2 Thirteen Features were Identified as Predictors, with no Baseline Differences in Training and Test Sets

After preprocessing the dataset variables, we ultimately retained 54 as candidate variables for feature selection. These candidates encompassed various categories: four demographic characteristics (age, sex, area, and insurance), 23 medical histories (smoking, hypertension, previous ischemic stroke, etc.), five medications prior to admission (antiplatelet, anticoagulation, diabetic medication, etc.), four arrival and admission information (time from onset to door, National Institutes of Health Stroke Scale (NIHSS) on presentation, hospital level, etc.), 14 physical and laboratory examinations after admission (weight, blood pressure, platelet, fasting glucose, etc.), and four in‐hospital treatment and complications (antiplatelet, intravenous t‐PA thrombolytic, dysphagia assessment results, etc.). Among the 54 variables outlined in Table S1, 185,774 cases exhibited no missing values. Of these, 8536 were patients who experienced in‐hospital stroke recurrence. Following downsampling (2:1 ratio to positive cases) for the negative cases, 17,072 patients without recurrence were retained. Post‐downsampling comparisons (Table S2) revealed no substantial shifts in baseline characteristics (standardized mean difference [SMD] < 0.1 for 52 out of 54 variables), except for higher prevalence of previous ischemic stroke and antiplatelet medication history in positive cases, with these differences attributable to their established prognostic roles in stroke recurrence as shown in Table S1 rather than sampling artifacts. MLR was then performed on the datasets obtained from three random draws. Ultimately, 13 feature variables were kept as final participant predictors, including age, previous ischemic stroke, previous intracerebral hemorrhage, carotid artery stenosis history, heart failure history, peripheral vascular disease (PVD) history, antiplatelet medication history, NIHSS on presentation, systolic blood pressure, international normalized ratio, hemoglobin A1C, homocysteine, and assessment of dysphagia upon admission. The top three strong predictors, ranked by odds ratio (OR), were a history of previous ischemic stroke, heart failure, and PVD. The most significant effect was observed for previous ischemic stroke. Table 1 presents the specific effects of all 13 features on the outcome variables, along with baseline information for each feature in both the training and test sets. No significant differences were observed between the training and test sets, with all predictors exhibiting an SMD below the threshold of 0.1.

**TABLE 1:** Final participant predictors determined by MLR and the baseline characteristics in training and test sets.

	Training set (*n* = 221,976)	Test set (*n* = 100,159)	SMD	OR	95% CI
Demographics					
Age, median [IQR]	66 [57;74]	65 [57;74]	0.011	1.005	1.001–1.010
Medical history					
Previous ischemic stroke, *n* (%)	64794 (29.5%)	27417 (27.6%)	0.042	7.849	7.032–8.769
Previous ICH, *n* (%)	4973 (2.2%)	2116 (2.1%)	0.009	1.397	1.038–1.878
Carotid artery stenosis, *n* (%)	3027 (1.4%)	1182 (1.2%)	0.016	1.436	1.038–2.002
Heart failure, *n* (%)	1728 (0.8%)	656 (0.7%)	0.015	1.702	1.066–2.721
PVD, *n* (%)	3771 (1.7%)	1379 (1.4%)	0.027	1.610	1.162–2.249
Medications prior to admission					
Antiplatelet, *n* (%)	45,829 (21.3%)	20,690 (21.2%)	0.005	1.471	1.311–1.651
Arrival and admission information					
NIHSS on presentation			0.022	1.052	1.018–1.088
0, *n* (%)	29,228 (13.2%)	12,655 (12.6%)			
1, *n* (%)	40,519 (18.3%)	18,437 (18.4%)			
2, *n* (%)	55,477 (25.0%)	24,744 (24.7%)			
3, *n* (%)	40,264 (18.1%)	18,749 (18.7%)			
4, *n* (%)	34,431 (15.5%)	15,453 (15.4%)			
5, *n* (%)	22,057 (9.9%)	10,121 (10.1%)			
Physical and laboratory examination					
Systolic blood pressure (mmHg), median [IQR]	149 [134;162]	149 [135;163]	0.016	1.003	1.001–1.005
INR, median [IQR]	0.99 [0.93;1.06]	1 [0.94;1.07]	0.024	1.077	1.015–1.144
Hemoglobin A1C (%), median [IQR]	5.8 [5.2;6.6]	5.8 [5.3;6.6]	0.015	1.042	1.011–1.073
HCY (µmol/L), median [IQR]	13.8 [10.3;19.2]	13.72 [10.2;19.0]	<0.001	1.004	1.000–1.008
In‐hospital treatment and complications					
Dysphagia, *n* (%)	8767 (4.6%)	4076 (4.8%)	0.009	1.531	1.250–1.874

Abbreviations: CI, confidence interval; HCY, homocysteine; ICH, intracerebral hemorrhage; INR, international normalized ratio; IQR, interquartile range; MLR, multivariate logistic regression analysis; NIHSS, National Institutes of Health Stroke Scale; OR, odds ratio; PVD, peripheral vascular disease; SMD, standardized mean differences.

### 2.3 All Machine Learning Models Outperformed the Traditional Generalized Linear Model

**FIGURE 2:**
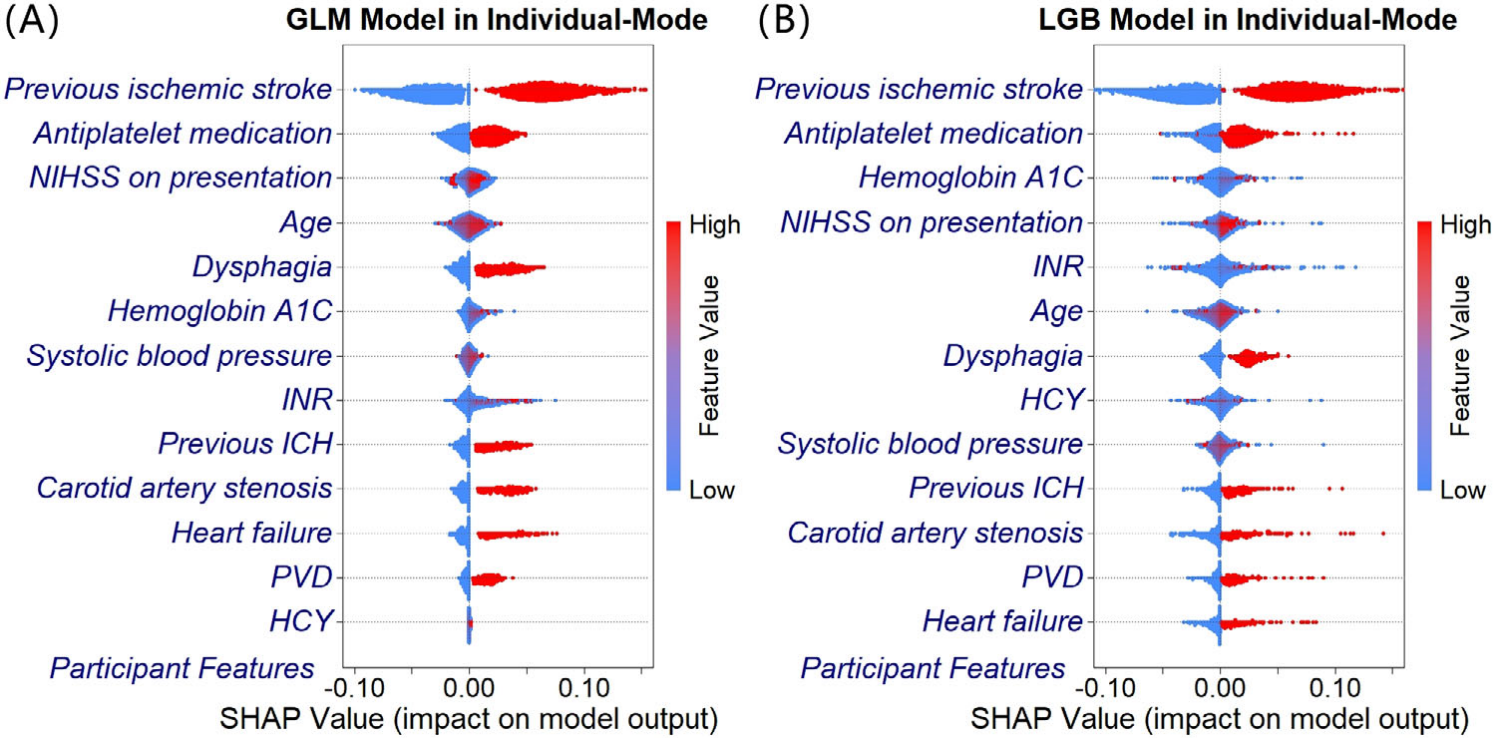
The Shapley additive explanations values of the benchmark model (GLM) (A) and the best prediction model (LGB) (B) in individual‐mode in training set. *Abbreviations*: GLM, generalized linear model; LGB, light gradient boosting; NIHSS, National Institutes of Health Stroke Scale; INR, international normalized ratio; HCY, homocysteine; ICH, intracerebral hemorrhage; PVD, peripheral vascular disease; SHAP, Shapley additive explanations. *Note*: The horizontal axis illustrates the SHAP contribution values of each feature to the model output for each case, while the vertical axis signifies the features involved in the perdition model. The histogram below the horizontal line of each feature displays the distribution of SHAP values for individuals who did not experience stroke recurrence during hospitalization, and the histogram above the line represents those who did. Both histograms are presented on a logarithmic scale. Colors depict the feature values for each case.

In the individual‐mode model's prediction of in‐hospital stroke recurrence among patients with acute minor ischemic stroke, the area under the curve (AUCs) for the test set were as follows: generalized linear model (GLM) (0.779, 95% confidence interval [CI]: 0.772–0.786), extreme gradient boosting (XGB) (0.788, 95% CI: 0.782–0.794), light gradient boosting (LGB) (0.789, 95% CI: 0.783–0.796), and adaptive boosting (ADA) (0.787, 95% CI: 0.781–0.794), detailed in Table 2. The *p* values for the three machine learning models compared with the GLM model were all less than 0.0001. However, the *p* values for XGB and ADA compared with LGB were 0.1268 and 0.0232, respectively. All machine learning models outperformed the traditional GLM model, but no significant differences were found among the machine learning models themselves. The Brier scores across models are close, ranging from 0.04318 to 0.04335, indicating excellent calibration and minimal differences in the alignment between predicted probabilities and true incidence. Figure 2 illustrates the Shapley additive explanations values (SHAP) of the GLM and LGB model for each feature variable in the training set [32, 33]. In the SHAP chart, the features were ordered from top to bottom based on their predictive contributions, from largest to smallest. The farther the points were from the centerline (i.e., the wider the horizontal distribution), the greater the influence of that feature on the model's output (which could be positive or negative); the more dispersed the low‐value (in blue color) and high‐value (in red color) points, the clearer the effect of the variable values on the prediction. Among the important variables indicated by the SHAP for GLM and LGB, five of the top six variables overlapped, including previous ischemic stroke, antiplatelet medication history prior to admission, NIHSS on presentation, age, and hemoglobin A1C, with a history of ischemic stroke and antiplatelet medication consistently occupying the top two positions. Unlike the OR values suggested by MLR, the importance of a history of heart failure and PVD ranked relatively lower in the SHAP plots.

**TABLE 2:** Diagnostic indicators of GLM, XGB, LGB, and ADA models in individual‐mode and combined‐mode for stroke recurrence prediction in the test set (30% of the centers included).

	Individual‐mode				Combined‐mode			
	GLM	XGB	LGB	ADA	GLM	XGB	LGB	ADA
AUC	0.779	0.788	0.789	0.787	0.781	0.794	0.803	0.788
	0.772–0.786	0.782–0.794	0.783–0.796	0.781–0.794	0.775–0.788	0.788–0.801	0.797–0.809	0.781–0.794
Youden Index	0.506	0.507	0.505	0.508	0.507	0.505	0.508	0.506
	0.493–0.518	0.494–0.518	0.493–0.517	0.495–0.520	0.495–0.520	0.492–0.516	0.496–0.520	0.493–0.518
SENS (%)	76.1	77.2	78.3	76.8	76.4	77.7	76.6	75.8
	74.9–77.3	76.1–78.3	77.2–79.5	75.6–78.0	75.2–77.7	76.5–78.9	75.4–77.7	74.6–76.9
SPEC (%)	74.5	73.5	72.2	73.9	74.3	72.7	74.3	74.8
	74.2–74.8	73.2–73.7	71.9–72.5	73.6–74.2	74.0–74.5	72.5–73.0	74.0–74.5	74.5–75.1
Brier score	0.04335	0.04322	0.04318	0.04319	0.04331	0.04317	0.04312	0.04321
*p* Value^a^	/	/	/	/	0.0021	<0.0001	<0.0001	0.6783
*p* Value^b^	/	<0.0001	<0.0001	<0.0001	/	<0.0001	<0.0001	<0.0001
*p* Value^c^	<0.0001	0.1268	/	0.0232	<0.0001	<0.0001	/	<0.0001

Abbreviations: ADA, adaptive boosting; AUC, area under ROC curve; GLM, generalized linear model; LGB, light gradient boosting; ROC, receiver operation characteristic; SENS, sensitivity; SPEC, specificity; XGB, extreme gradient boosting.

^a^ The *p* values were calculated by the Delong AUC Difference Test for each model across two modes.

^b^ The *p* values were calculated by the Delong AUC Difference Test for each machine learning model compared with GLM.

^c^ The *p* values were calculated by the Delong AUC Difference Test for each machine learning model compared with LGB.

### 2.4 LGB Model Emerged as the Top Performer, Especially After Optimization

**FIGURE 3:**
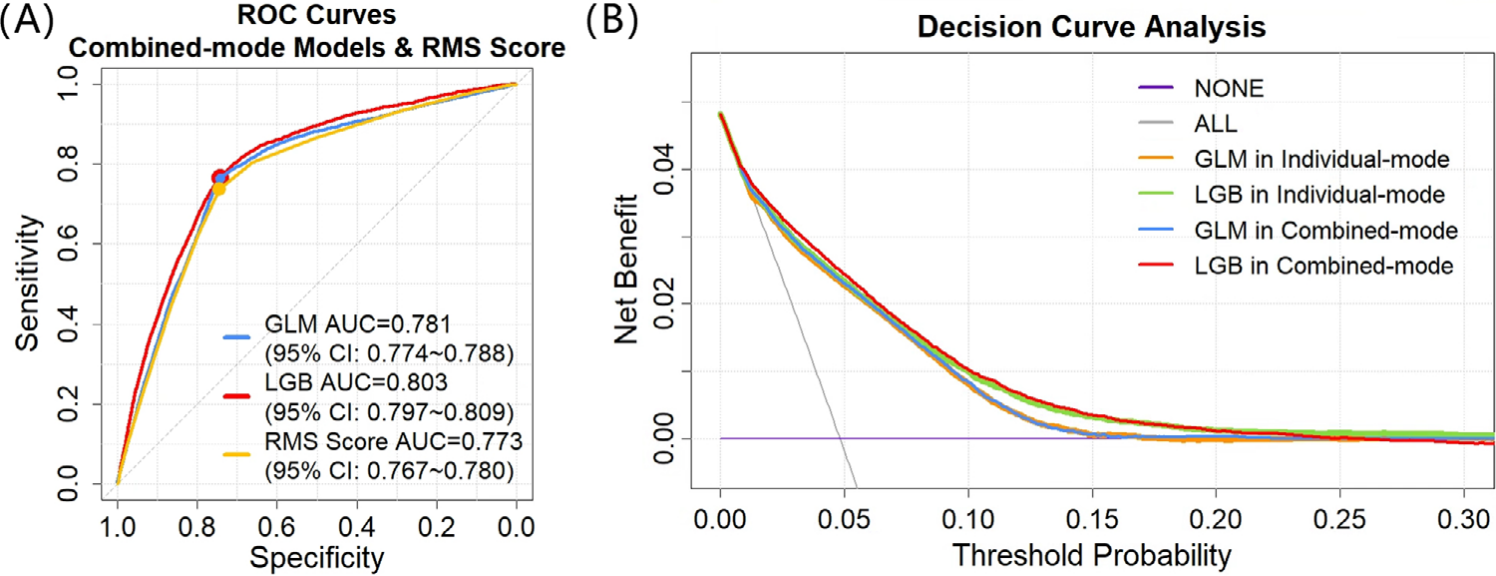
Predictive performance evaluation for the developed GLM model, machine learning model and scoring scale: (A) the ROC curves for the GLM and LGB models in combined‐mode and RMS score in the test set; (B) the decision curves for the default strategies and for the GLM and LGB models before and after combinatorial optimization in the test set. *Abbreviations*: GLM, generalized linear model; LGB, light gradient boosting; RMS, in‐hospital stroke recurrence prediction scale for patients with acute minor ischemic stroke; AUC, area under ROC curve; ROC, receiver operation characteristic; CI, confidence interval.

Following combinatorial optimization, both the XGB and LGB models exhibited varying degrees of improvement in AUC, with the *p* values compared with their preoptimization individual‐mode model being less than 0.0001. While the GLM and ADA models showed slight changes in AUC, despite the GLM demonstrated statistical significance (*p* < 0.05), whereas the ADA model, with a *p* value of 0.6783, indicated no significant difference. Among them, the LGB model demonstrated a significant enhancement, outperforming not only the models prior to optimization but also the other two machine learning models postoptimization. The AUC of each model after optimization was as follows: GLM 0.781 (95% CI: 0.775–0.788), XGB 0.794 (95% CI: 0.788–0.801), LGB 0.803 (95% CI: 0.797–0.809), and ADA 0.788 (95% CI: 0.781–0.794) respectively. The receiver operating characteristic (ROC) curves in Figure 3A depicts the performance of the GLM and LGB model after combinatorial optimization. Figure 3B illustrated the decision curves [34, 35, 36] in test set for the LGB models before and after combinatorial optimization, in comparison with the GLM model. The results reveal that the LGB model offers a higher net benefit than the GLM model and default strategies of “treat all” or “treat none” across the entire reasonable range of threshold probabilities. In summary, all machine learning models (XGB, LGB, and ADA) exhibited superior performance in predicting in‐hospital stroke recurrence among patients with acute minor ischemic stroke, significantly outperforming traditional GLM methods. Notably, the LGB model emerged as the top performer, especially after optimization using the combined‐mode approach. This optimization resulted in superior discrimination, achieving the highest AUC of 0.803, and robust calibration, with the lowest Brier score of 0.04312.

### 2.5 The Model's Predictive Performance is Consistent Across Different Subgroups

**FIGURE 4:**
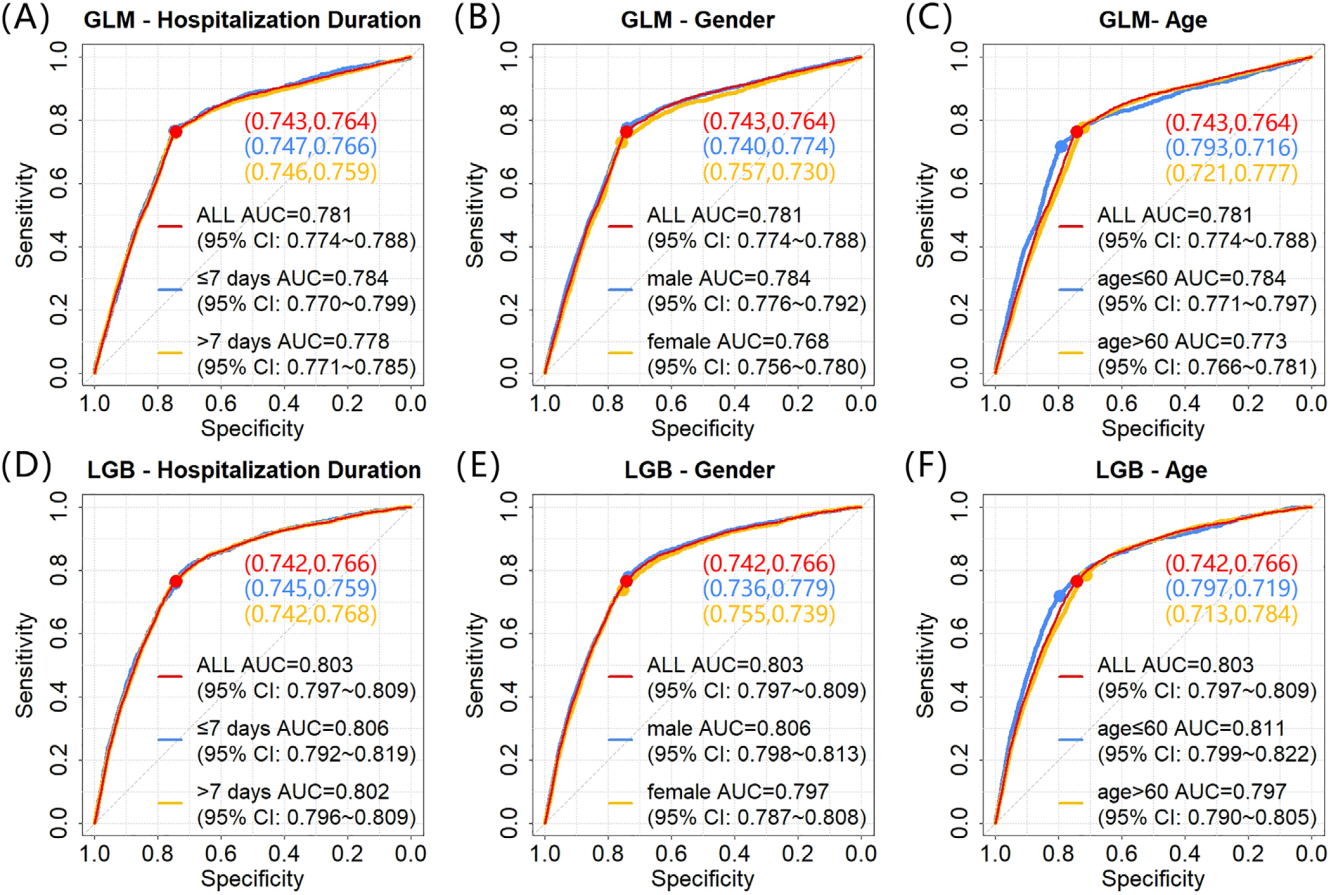
Model performance of the GLM (A, B, and C) and LGB (D, E, and F) models after combinatorial optimization in the test set, on subgroups based on hospitalization duration (A and D), gender (B and E), and age (C and F). Legend: (specificity, sensitivity). *Abbreviations*: GLM, generalized linear model; LGB, light gradient boosting; AUC, area under ROC curve; ROC, receiver operation characteristic; CI, confidence interval.

Stroke recurrence is closely related to the duration since onset, shorter intervals from onset are associated with a higher likelihood of recurrence. We aimed to analyze subgroup of patients experienced stroke recurrence within a fixed time period. However, due to the lack of data on the time interval from admission to recurrence in the CSCA database, we could only conduct a secondary analysis of recurrence within different lengths of hospital stay, 7 days or less versus more than 7 days. We have also conducted the same analysis for subgroups based on gender and age. Figure 4 exhibits the ROC curves of the combined‐mode GLM and LGB model across different subgroups of the test set, along with the sensitivity and specificity at the optimal thresholds determined by the training set ROC curve for each subgroup in the test set. Further results from the subgroup analysis of the test set, including the XGB and ADA models after combinatorial optimization as well as each model before optimization, can be found in Figures S1–S3. The results indicate that the predictive capability of the model is similar across various subgroups, particularly between the subgroups for length of hospital stay and gender. In terms of age subgroups, the subgroup aged 60 years or younger exhibited higher specificity, while the subgroup aged over 60 years demonstrated higher sensitivity.

### 2.6 A Clinical Easy‐to‐Use Scale was Developed, with Performance Slightly Lower than GLM but Still Quite Good

To integrate the findings of this study into clinical practice, one approach is to develop the LGB model as a standalone software tool that allows clinicians to input the relevant predictive variables and receive real‐time recurrence predictions. Another approach is to convert the model into a scoring scale. For this purpose, we incorporated the final participant predictors into the scoring system, assigning points to each variable based on their OR values from the MLR analysis (Table 1). The scoring thresholds for laboratory examinations, age, NIHSS, and systolic blood pressure were determined by considering their normal reference ranges alongside the optimal thresholds of each variable's ROC curve for predicting in‐hospital stroke recurrence (detailed in Figure S4). The strongest predictor, previous stroke history, was assigned a point of 4, as the AUC of the scoring scale began to plateau beyond from this point. A 13‐item in‐hospital stroke Recurrence prediction scale for patients with acute Minor ischemic Stroke (RMS) was created, with a total score ranging from 0 to 16, as shown in Table 3. Patients with a score of 6 or higher were at a higher risk of stroke recurrence during hospitalization. The ROC curve of the RMS score in the test set is shown in Figure 3A, with an AUC of 0.773, a specificity of 0.744 and a sensitivity of 0.737 at the optimal threshold. While the LGB model achieves higher discriminative performance, its implementation requires specialized software and computational resources that may not be available in all clinical settings. Conversely, the RMS score can be readily used without additional software, enabling clinicians to rapidly assess stroke recurrence risk using a straightforward scoring system. The RMS score prioritizes clinical usability over maximal predictive accuracy, offering a practical alternative to the LGB model.

**TABLE 3:** Individual components of the RMS score.

Variables	Criteria	Points
Demographics		
Age	≤60	0
	>60	1
Medical history		
Previous ischemic stroke	Absent	0
	Present	4
Previous ICH	Absent	0
	Present	1
Carotid artery stenosis	Absent	0
	Present	1
Heart failure	Absent	0
	Present	1
PVD	Absent	0
	Present	1
Medications prior to admission		
Antiplatelet	Absent	0
	Present	1
Arrival and admission information		
NIHSS on presentation	≤2	0
	>2	1
Physical and laboratory examination		
Systolic blood pressure	≤140 mmHg	0
	>140 mmHg	1
INR	≤1	0
	>1	1
Hemoglobin A1C	≤6.5%	0
	>6.5%	1
HCY	≤15 µmol/L	0
	>15 µmol/L	1
In‐hospital treatment and complications		
Dysphagia	Absent	0
	Present	1

Abbreviations: HCY, homocysteine; ICH, intracerebral hemorrhage; INR, international normalized ratio; NIHSS, National Institutes of Health Stroke Scale; PVD, peripheral vascular disease.

## 3 Discussion

In the stroke registry cohort CSCA established on the national hospital‐based stroke care quality assessment and improvement platform, we developed and validated a combined‐mode machine learning model for the prediction of stroke recurrence during hospitalization in patients with acute minor ischemic stroke.

When comparing machine learning prediction models to those constructed using traditional GLM, the findings indicate that some machine learning models outperformed GLM in predicting stroke recurrence during hospitalization in patients with acute minor ischemic stroke. Among them, the LGB model demonstrated the highest AUC and the lowest Brier score, particularly after the optimization of the combined model. However, it should be noted that not all machine learning models surpassed the GLM model. In our preliminary experiments, the support vector machine (SVM) model had the lowest modeling efficiency and performed worse than the GLM model in predictive accuracy. Additionally, the random forest (RF) model exhibited a tendency to overfit the training set, which compromised its generalization ability and led to mediocre performance in the test set. The high computational complexity of the SVM model and the erratic overfitting behavior of RF model made them unsuitable for the prediction scenarios in this study.

Comparatively, both the XGB and LGB models excel at controlling the issue of overfitting. During the model tuning process, they maintain a good balance between model performance and the number of training iterations, preventing the occurrence of overfitting problems. Among them, the performance of the LGB model is the best. Compared with the XGB and ADA models, it achieves superior predictive performance in shorter training iteration times and with less resource consumption.

In this study, the commonly used traditional GLM model exhibits stable performance and already achieves good accuracy in predicting stroke recurrence. The utilization of machine learning models, especially those optimized through combinatorial methods, has further significantly enhanced the predictive accuracy. Moreover, the predictor variables input into the model are the most common in clinical practice and routinely documented in the management of stroke patients. The accessibility of these variables makes the machine learning prediction model a good potential for clinical application.

In the procedure of feature selection and model development, this study identified 13 predictor variables. These are well established and recognized stroke risk factors, including medical history related to stroke, cerebrovascular risk factors like age, hypertension, hyperglycemia, as well as neurological deficits and complications such as dysphagia during this episode of stroke. In many previously established models, age and blood pressure were both adopted as predictive factors [15, 20]. In stroke recurrence risk prediction models developed for ischemic stroke patients, a history of ischemic stroke was also adopted as a predictive factor [15, 20]. In the FSRJ [21] and ESRS [22, 23] prediction models, the predictive factors included myocardial infarction, atrial fibrillation, cardiovascular diseases, and peripheral arterial disease, which are similar to the history of heart failure and PVD included in our study. While, hyperglycemia is widely used as a predictor in scoring systems other than RRE‐90 [24].

Among these predictive factors, the history of previous ischemic stroke has a significantly higher risk contribution level compared with other factors, reflected by a very high OR of 7.849. To mitigate overfitting and facilitate a more balanced model, the inclusion of medical history allowed for a prompt attainment of moderate performance. However, this approach may inadvertently restrain the predictive capacity of other feature variables beyond medical history. To address this limitation, the study proposes the construction of a stroke recurrence prediction model during hospitalization specifically for population experiencing their first stroke occurrence without the variable of previous ischemic stroke. This model is then optimized in combination with the overall population prediction model. Remarkably, the LGB model exhibited substantial and statistically significant performance enhancement. This improvement is possibly attributed to the further exploitation of predictive contributions from variables beyond previous stroke history in the combined‐mode model.

This study also has several limitations that warrant consideration.

First, the SVM and RF models exhibited suboptimal performance, and a thorough investigation into the reasons behind their ineffectiveness was not undertaken. Consequently, the specific factors rendering these models unsuitable for the prediction scenarios in this study remain unexplored.

Second, to the best of our knowledge, no studies have specifically focused on in‐hospital stroke recurrence. Since our method differs significantly from existing scoring methods in target population, predictor variables and predictive outcome (the time point for recurrence prediction), a direct head‐to‐head performance comparison is not feasible; nevertheless, comparative analyses with some previously reported methods are provided in Discussion 2 (Supporting Information) for reference. For example, methods applied to ischemic stroke patients (not limited to minor stroke) like FSRJ [21] and ESRS [22, 23] predict recurrence within 1 year, while RRE‐90 [24] predicts recurrence within 3 months. Among methods for TIA patients, the California score [37] predicts recurrence within 3 months, and although the ABCD series can predict recurrence within 2 and 7 days, it is limited to TIA patients [28, 38–41]. The ABCD^2^‐MRI score [27], applicable to minor stroke patients, predicts recurrence within 3 months. Methods developed for mixed populations of TIA and minor stroke patients, such as SPI‐I [25], SPI‐II [26], Dutch TIA [29], and LiLAC [30], are used to predict recurrence over 2–10 years. Therefore, in our study, the model's performance was evaluated on the test set created by stratifying data from different centers. This strategy allowed for an external‐like validation within our current available CSCA database, as no centers overlapped between the training and test sets. While this method offers partial evidence of external validation and supports the model's generalizability, a more robust evaluation would ideally involve an independent, multicenter, or prospective cohort. Although the CSCA is a multicenter cohort with a certain degree of broad representation in terms of hospitals, patients, and regions, the participating hospitals are more likely to be larger, tertiary centers with a multitude of resources that smaller hospitals do not have access to. Utilizing external datasets that are more representative of hospital distribution can further validate the model and enhance its generalizability.

Third, in this study, GLM was employed as a benchmark to compare the performance of three machine learning models in predicting in‐hospital recurrence among patients with minor stroke. To ensure fairness across models, we applied same criteria for feature selection, avoiding machine learning‐specific feature selection methods such as Boruta [42] or SHAP [32, 33]. Instead, traditional MLR was used to select the feature variables, which were then consistently applied to all models. While this approach ensures better interpretability, it may have constrained the potential for further improving the predictive performance of the machine learning models. However, supplemental analyses exploring Boruta‐based feature selection (Discussion 1, Supporting Information) revealed no actual performance gains. Models trained on machine learning‐derived features showed reduced discrimination and poorer calibration compared with those using features selected by logistic regression. This suggests that while machine learning‐based feature selection can identify important predictors, logistic regression remains a robust method for selecting features that maintain high model performance and clinical interpretability.

Fourth, this study is constrained by insufficient temporal granularity in the CSCA registry. While analyzing temporal risk dynamics (e.g., daily risk evolution during hospitalization) would provide critical clinical insights, such investigations are precluded by systematically absent temporal metadata. Specifically, the registry lacks: precise timing of recurrent strokes (postadmission day/hour), distinction between single versus multiple recurrence events, and time‐stamped clinical parameter trajectories. This data limitation directly impacted our subgroup analysis methodology. We originally planned to stratify patients by recurrence timing since stroke onset (e.g., ≤7 days vs. >7 days). However, as recurrence timestamps were unavailable, we pragmatically adopted hospitalization duration as a surrogate grouping criterion. While this approach allows confirming recurrence within ≤7 days for short‐stay patients, it introduces ambiguity for those hospitalized >7 days (inability to distinguish early versus late in‐hospital recurrence). Although no significant rate differences emerged between stay duration subgroups, this analytical compromise underscores the need for future time‐resolved datasets to validate the model's temporal robustness. This temporal ambiguity also precluded systematic recording of NIHSS scores and imageological data at stroke recurrence timepoints, thereby preventing detailed analysis of new infarct lesions or neurological deterioration severity during recurrent events.

Moreover, the etiology of ischemic stroke classified by Trial of Org 10172 in Acute Stroke Treatment (TOAST) criteria is highly relevant to stroke recurrence [12]. However, since TOAST classification is generally determined during hospitalization after completing all necessary examinations, not at the time of patient admission, it cannot be incorporated into our predictive model for estimating the risk of in‐hospital recurrence at admission. Additionally, several crucial predictors considered in this study for constructing the model were nonmodifiable variables, such as age, history of stroke, cerebrovascular disease, heart disease, and previous medication history. Dysphagia at admission was identified as a significant risk factor for stroke recurrence during hospitalization, but it remains a challenging variable to modify. While other vascular risk factors, such as hypertension and homocysteine levels, were associated with stroke recurrence, their impact appeared modest. Furthermore, there is limited evidence that controlling these modifiable risk factors would lower the stroke recurrence during the hospital stay.

The significance of this study lies in its pioneering effort to predict high‐risk patients for stroke recurrence during hospitalization using machine learning methods. It serves as a reminder for clinicians to conduct a thorough and comprehensive assessments at an appropriate time point after a patient's admission, enabling the early selection of more targeted therapeutic options to reduce the risk of stroke recurrence. However, this study did not investigate the potential intervening factors that could reduce in‐hospital recurrence in patients with acute minor ischemic stroke, nor did it examine how interventions might achieve this reduction. The strategies recommended by the 2019 AHA/ASA Guidelines [43] and related high‐impact studies [6, 13, 19] include (1) intensified dual antiplatelet therapy, (2) early personalized secondary prevention, (3) enhanced vascular evaluation, and (4) early rehabilitation. Future research should focus on controllable variables in the treatment process to identify effective interventions for reducing stroke recurrence during hospitalization in this patient population.

## 4 Conclusion

For the first time, this study has created a model specifically designed to predict in‐hospital stroke recurrence in patients with acute minor ischemic stroke. The machine learning prediction model demonstrated superior accuracy compared with traditional GLM methods. Notably, the LGB model exhibited remarkable performance, especially after the optimization of the combined model. The predictive capabilities of the model warrant further validation in external or prospective cohorts in future studies. Moreover, incorporating more detailed variables related to the in‐hospital therapeutic process could enhance the identification of intervention strategies aimed at reducing stroke recurrence during hospitalization in these patients.

## 5 Methods

### 5.1 Study Design and Population

The study population was derived from data within the CSCA database, a prospective, national, hospital‐based, multicenter, voluntary, multifaceted intervention program focused on evidence‐based performance measurement monitoring and feedback [44]. Initiated by the China Stroke Association in June 2015, the program aimed to standardize and enhance the quality of stroke care in China. From August 1, 2015 to July 31, 2019, 1471 hospitals in China contributed to the CSCA program, and a total of 1,006,798 patients were prospectively enrolled. The CSCA program adhered to the Declaration of Helsinki principles. Ethical clearance was acquired from the ethical review committee of Beijing Tiantan Hospital (ethical approval number: KY2018‐061‐02). The requirement for explicit informed consent was exempted by their institutional review board.

Inclusion criteria for this study were as follows: (1) individuals aged 18 years or older; (2) final diagnosis of acute ischemic stroke at discharge confirmed by brain CT or MRI [44]; (3) NIHSS of 5 or less at initial presentation. Exclusion criteria encompassed cases where the outcome of in‐hospital stroke recurrence was missing. In the CSCA database, in‐hospital stroke recurrence is defined as encompassing ischemic stroke, hemorrhagic stroke, and symptomatic hemorrhagic transformation of ischemic stroke, while excluding TIA recurrence.

### 5.2 Database Variable Preprocessing

The CSCA database contained a total of 545 variables, and the outcome variable for this study was in‐hospital stroke recurrence in patients with acute minor ischemic stroke. Among the variables preceding the recurrence event, we screened variables by drawing upon clinical expertise, guidelines for early management of acute ischemic stroke [43], relevant predictive models from the literature [45, 46], and the unique characteristics of the CSCA data. Additionally, we excluded variables with more than 30% missing data, ultimately identified the candidate variables for feature selection. Patients were categorized into two groups based on the outcome variable (those with in‐hospital stroke recurrence and those without) to analyze the baseline characteristics of the candidate variables.

### 5.3 Model Development

#### 5.3.1 Feature Selection

Given the study's large sample size, there exists a potential pitfall wherein variables may exhibit statistical significance yet lack genuine predictive power. Reducing the number of predictors can enhance computational efficiency and improve model interpretability. To address this, we employed a conventional multivariate binomial logistic regression with stepwise‐backward procedure based on the likelihood ratio test to identify the most relevant subset from candidate features. These features (predictors) were then used in the modeling each subsequent machine learning model. The specific approach was as follows. First, cases with no missing values across the candidate variables listed in Table S1 were curated, forming the dataset for feature selection. Next, to mitigate potential bias introduced by class imbalance in logistic regression, downsampling of negative class (patients without in‐hospital stroke recurrence) over positive class (patients with in‐hospital stroke recurrence) was conducted at a 2:1 ratio. Random sampling was then performed three times, selecting 15% of cases for MLR during each iteration. Variables that remained in the regression equation after each iteration were ultimately consolidated into a union set. Finally, based on the consolidated dataset derived from these three random samples, another round of MLR was conducted to identify the final set of predictors.

#### 5.3.2 Imputation of Missing Values

Multivariate imputation by chained equations was used to impute missing data due to its flexibility with various variable types, its ability to preserve variable correlations for more accurate estimates [47]. Specifically, predictive mean matching was employed to impute missing values for numerical continuous variables, a proportional odds model was utilized for categorical variables with hierarchical meanings, and polytomous logistic regression was used for categorical variables without hierarchical meanings. Furthermore, to manage continuous variables with wide‐range fluctuating values in laboratory data, a deviation standardization method was applied to scale the data to a uniform range between 0 and 1 through linear transformation.

#### 5.3.3 Training and Test Sets

The dataset after imputation of missing values was randomly split into a training set and a test set according to the different centers, with the ratio of center numbers being 7:3. Fivefold cross‐validation on the training set were employed to optimize hyperparameters, subsequently retraining the model on the entire training set and evaluating it on the test set. The optimal threshold, determined by the maximizing the Youden index in the ROC curve of the training set, was applied to the test set to evaluate the classification performance of the prediction model. Furthermore, we utilized SHAP to express the feature attribution numerically. The visual representation of the SHAP chart allows for an intuitive understand the impact of feature variable values on the prediction outcome.

#### 5.3.4 Generalized Linear Model

GLM is a classical linear model that is commonly employed in classification scenarios. It operates by mapping the output of a linear regression model into a range between 0 and 1. Predictions are made by inputting data into a pretrained logistic regression model, and classification decisions are based on the resulting probability values [48]. In this study, GLM model served as a benchmark, due to its simplicity and ease of interpretation, for evaluating the performance of other machine learning models.

#### 5.3.5 Selection of Machine Learning Models

We evaluated and compared the strengths and weaknesses of five machine learning algorithms: SVM, RF, XGB, LGB, and ADA.

The SVM model, introduced by Cortes and Vapnik [49], was extended to the nonlinear domain by Boser et al. [50] adding the kernel trick. Renowned for its generalization ability and robustness, SVM excels in handling small samples and high‐dimensional data but heavily depends on the careful selection of hyperparameters. It is also computationally expensive, especially for large datasets [51]. In our study, due to the large scale of the study cohort, the SVM modeling was extremely time‐consuming, with each training iteration taking about 48 h. This inefficiency made hyperparameter tuning impractical, especially with fivefold cross‐validation. Given these issues, SVM is unsuitable for this study and will be excluded from further consideration.

RF is an ensemble learning method that combines the predictions of multiple decision trees to improve accuracy [52, 53]. RF can handle a large number of input features and is less sensitive to outliers, but it may require more computational resources due to the construction of multiple trees. In our pre‐experiment, RF was highly prone to overfitting in the training set, which rendered the optimal classification threshold determined during training unsuitable for the test set. Reducing overfitting through parameter tuning improved the consistency of thresholds between the training and test sets, but this came at the cost of a significant decrease in AUC for the test set. Due to these challenges, RF is not well suited for this study and will not be pursued further.

XGB is a powerful ensemble method known for high predictive accuracy and handling nonlinear data. XGB addresses overfitting by controlling model complexity through second‐order Taylor expansion of the loss function and the introduction of a regularization term [54, 55]. It demonstrated efficient modeling capabilities in our pre‐experiments without significant overfitting.

ADA is another ensemble technique that adjusts the weights of misclassified instances, improving accuracy over iterations. While sensitive to noisy data and slower in modeling [56], its performance was within acceptable limits during pre‐experiments, warranting its selection.

The LGB model, based on the gradient boosting decision tree algorithm [57], incorporates techniques such as feature bundling, histogram algorithms, and depth‐first growth. LGB showcases faster training speed, lower memory consumption, and excels in efficiently handling large‐scale data and high‐dimensional features.

After careful evaluation and comparison, XGB, ADA, and LGB were ultimately chosen as the final models for this study.

### 5.4 Model Optimization

**FIGURE 5:**
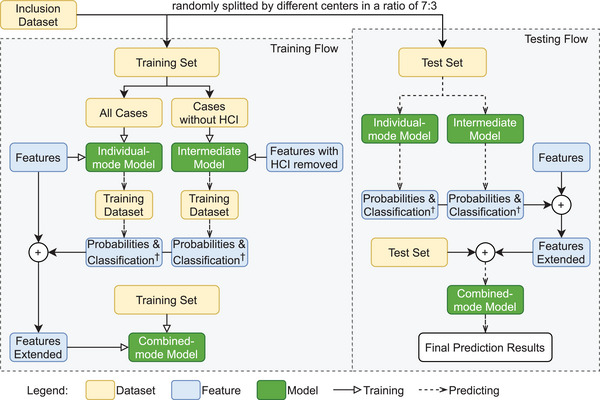
Block diagram of model training and testing processes for both individual‐mode and combined‐mode. Abbreviations: HCI, previous history of cerebral ischemic stroke. Created using draw.io‐desktop version 14.6.13. ^†^The threshold for predicting stroke recurrence during hospitalization in the test dataset is maintained consistent with that identified in the training dataset's ROC curve, which is determined by maximizing the Youden index.

Considering that the prominent contribution of the “previous ischemic stroke” among the feature variables might have disproportionately influenced the model's attention, we were concerned that this dominant variable might prematurely halt the model's iterations, potentially introducing prediction bias in the majority of patients without a history of previous ischemic stroke and diminishing the impact of the other variables in predicting recurrence. The model framework was adjusted to enhance its performance within this subgroup. In addition to the individual training model (referred to as the individual‐mode model), which included all features, we introduced an intermediate model that excluded “previous ischemic stroke” from the feature set and was trained only on cases without a history of ischemic stroke. This adjustment aimed to better capture the predictive power of other features. The predicted probabilities from both the individual‐mode and intermediate models on the training set, along with the classification results determined by the threshold derived from maximizing Youden index in the ROC curve, were amalgamated as a new variable. This derived variable, in conjunction with the original feature variables, constituted an extended feature set utilized in developing the final combinatorial optimization model on the training set, termed the combined‐mode model. The individual‐mode, intermediate, and combined‐mode models differ only in the inclusion of stroke history as a feature, and the training populations were modified accordingly. Model parameters were optimized using fivefold cross‐validation across all models. When evaluating the new model, the same procedure as the training process was employed to construct the new extended input variables and then to perform the evaluation procedure using the combined‐mode model on the test set. The discrimination thresholds used for each model (individual‐mode, intermediate, combined‐mode) in testing stage were aligned with those of the corresponding models in the training set. Figure 5 provides the block diagram of model training, testing and optimization.

### 5.5 Statistical Analysis

Continuous measurement variables were represented using medians with an interquartile range. Categorical variables were presented as numbers and frequencies. All data have been confirmed to be non‐normal in distribution. Due to the large sample size of our study, conventional tests like the Wilcoxon rank‐sum test or Chi‐square test could easily yield statistically significant differences that lack clinical meanings. Therefore, we opted to use SMD to compare baseline group differences between those with and without in‐hospital stroke recurrence [58], as well as between training and test sets. Where an SMD > 10% were considered meaningful [59, 60]. During model evaluation, the outcome variable was used as the dependent variable, while the predictor variables served as the independent variables in the training set. The model's performance was evaluated using discrimination metrics (AUC, Youden index, sensitivity, and specificity) and calibration metrics (Brier score), calculated from ROC curves and probabilistic accuracy assessments, respectively. The Delong paired ROC test was utilized to compare AUC differences among the four models. The corresponding 95% CIs were calculated using 2000 stratified bootstrap replicates, ensuring robust and accurate estimates. Two‐tailed *p* values of less than 0.05 in Delong ROC test were interpreted as statistically significant.

All statistical analyses, including the feature selection via MLR, the development of the GLM model and three machine learning models, as well as the visualization of results, were conducted using R 4.2.2 (The R Project for Statistical Computing. R Foundation. https://www.R‐project.org/).

## Author Contributions

Zixiao Li and Yongjun Wang contributed to the conception of the study, clinical expertise, and guidance of methodology for the study. Wanxing Ye and Jin Gan prepared the data set for usage and established the machine learning models. Ziyang Liu provided guidance for the model construction and fine tuning. Meng Wang provided guidance for feature selection and statistical analysis. Wanxing Ye conducted analysis of the results and statistical analysis. Wanxing Ye and Jin Gan drafted the original version of the manuscript and designed the figures and tables. Hongqiu Gu, Xin Yang, Chunjuan Wang, Xia Meng, Yong Jiang, Hao Li, and Liping Liu contributed to the acquisition of data. The corresponding authors attest that all listed authors meet authorship criteria and have full access to all the data in the study and take responsibility for the integrity of the data and the accuracy of the data analysis. All authors have read and approved the final manuscript.

## Conflicts of Interest

The authors declare no conflicts of interest.

## Ethics Statement

The CSCA program adhered to the Declaration of Helsinki principles. Ethical clearance was acquired from the ethical review committee of Beijing Tiantan Hospital (ethical approval number: KY2018‐061‐02). The requirement for explicit informed consent was exempted by their institutional review board.

## Supporting information



Supporting Information

## Data Availability

The data for this study are available by contacting the corresponding author upon reasonable request.
