# Supplementary material for: A Combined‐Mode Machine Learning Model for Predicting Stroke Recurrence During Hospitalization in Patients with Acute Minor Ischemic Stroke
Source: MedComm (2020). 2025 May 19;6(6):e70234. doi: 10.1002/mco2.70234 (PMC12086374; doi:10.1002/mco2.70234)
Supplement: Supplementary file 1 — Supporting Information [file MCO2-6-e70234-s001.docx]

# Supplementary Materials

## Title Page

### Title

A combined-mode machine learning model for predicting stroke recurrence during hospitalization in patients with acute minor ischemic stroke

### Running Head

predictive model for in-hospital stroke recurrence

### Authors

Wanxing Ye^1,#^; Jin Gan^2,#^; Meng Wang^1,3^; Ziyang Liu^4^; Hongqiu Gu^1,3^; Xin Yang^1,3^; Chunjuan Wang^1,3^; Xia Meng^1,2,3,5,6,7^; Yong Jiang^1^; Hao Li^1,2,3,5,6,7^; Liping Liu^1,2^; Yongjun Wang^1,2,3,5,6,7,*^; Zixiao Li^1,2,3,5,6,7,*^

Affiliation:

^1^China National Clinical Research Center for Neurological Diseases, Beijing Tiantan Hospital, Capital Medical University, Beijing 100071, China

^2^Beijing Tiantan Hospital, Capital Medical University, Beijing 100071, China

^3^National Center for Healthcare Quality Management in Neurological Diseases, Beijing Tiantan Hospital, Capital Medical University, Beijing 100071, China

^4^Beijing Advanced Innovation Center for Biomedical Engineering, School of Biological Science and Medical Engineering, Beihang University, Beijing 100191, China.

^5^Advanced Innovation Center for Human Brain Protection, Capital Medical University, Beijing 100071, China

^6^Research Unit of Artificial Intelligence in Cerebrovascular Disease, Chinese Academy of Medical Sciences, Beijing 100071, China

^7^Center for Excellence in Brain Science and Intelligence Technology, Chinese Academy of Sciences, Shanghai 200031, China

^#^Wanxing Ye and Jin Gan contributed equally to this work.

^*^Corresponding authors.

### Email

Wanxing Ye: yewanxing@bjtth.org

Jin Gan: ganjin99@163.com

Meng Wang: wangmengpumc@163.com

Ziyang Liu: liuziyang1106@buaa.edu.cn

Hongqiu Gu: guhongqiu@yeah.net

Xin Yang: yangxin@ncrcnd.org.cn

Chunjuan Wang: chuner_wang@126.com

Xia Meng: mengxia45@163.com

Yong Jiang: jiangyong@ncrcnd.org.cn

Hao Li: lihao71@aliyun.com

Liping Liu: lipingsister@gmail.com

Yongjun Wang: yongjunwang@ncrcnd.org.cn

Zixiao Li: lizixiao2008@hotmail.com

### ORCID

Wanxing Ye: [https://orcid.org/](https://orcid.org/0009-0009-4718-0081)0009-0009-4718-0081

Zixiao Li: [https://orcid.org/](https://orcid.org/0009-0009-4718-0081)0000-0002-4713-5418

## Supplemental Tables

**Table S1** Baseline characteristics of patients in-hospital with minor ischemic stroke.

| **Characteristics** | **Patients without stroke recurrence**  **(n=306,403)** | **Patients with stroke recurrence**  **(n=15,732)** | **SMD** |
| --- | --- | --- | --- |
|  |  |  |  |
| **Demographics** |  |  |  |
| Age, median [IQR] | 65[56;74] | 67[60;75] | **0.161** |
| Sex, male, n (%) | 197572(64.5%) | 10439(66.4%) | 0.039 |
| Area |  |  | **0.230** |
| Eastern, n (%) | 78893(25.7%) | 3186(20.3%) |  |
| Southern, n (%) | 36271(11.8%) | 2054(13.1%) |  |
| Northern, n (%) | 63002(20.6%) | 4045(25.7%) |  |
| Central, n (%) | 59412(19.4%) | 3655(23.2%) |  |
| Northeast, n (%) | 27859(9.1%) | 1088(6.9%) |  |
| Southwest, n (%) | 29628(9.7%) | 1020(6.5%) |  |
| Northwest, n (%) | 11338(3.7%) | 684(4.3%) |  |
| Insurance |  |  | **0.121** |
| Urban Employee, n (%) | 94404(30.8%) | 5622(35.7%) |  |
| Urban Resident, n (%) | 58691(19.2%) | 2947(18.7%) |  |
| Rural Resident, n (%) | 122960(40.1%) | 5901(37.5%) |  |
| Self-pay, n (%) | 19180(6.3%) | 720(4.6%) |  |
| Others, n (%) | 11168(3.6%) | 542(3.4%) |  |
| **Medical History** |  |  |  |
| Smoking |  |  | **0.136** |
| No, n (%) | 180818(60.2%) | 9105(59.2%) |  |
| Quit, n (%) | 39065(13.0%) | 2696(17.5%) |  |
| Yes, n (%) | 80280(26.7%) | 3575(23.3%) |  |
| Drinking |  |  | 0.015 |
| Yes, n (%) | 75702(25.2%) | 3774(24.5%) |  |
| No, n (%) | 224786(74.8%) | 11611(75.5%) |  |
| Previous ischemic stroke, n (%) | 80462(26.5%) | 11749(75.1%) | **1.110** |
| Previous TIA, n (%) | 3900(1.3%) | 490(3.2%) | **0.128** |
| Previous SAH, n (%) | 815(0.3%) | 90(0.6%) | 0.047 |
| Previous ICH, n (%) | 6528(2.1%) | 561(3.6%) | 0.086 |
| Hypertension, n (%) | 197071(64.8%) | 11723(74.9%) | **0.221** |
| Diabetes mellitus, n (%) | 66004(21.8%) | 4477(28.7%) | **0.161** |
| Carotid artery stenosis, n (%) | 3622(1.2%) | 587(3.7%) | **0.165** |
| Atrial fibrillation, n (%) | 10166(3.4%) | 869(5.6%) | **0.109** |
| Heart failure, n (%) | 2126(0.7%) | 258(1.6%) | 0.088 |
| Prosthetic Heart Valve, n (%) | 379(0.1%) | 31(0.2%) | 0.018 |
| Sickle cell, n (%) | 13(0.0%) | 1(0.0%) | 0.003 |
| Current pregnancy or 6 week postpartum, n (%) | 30(0.0%) | 3(0.0%) | 0.008 |
| Dementia, n (%) | 934(0.3%) | 139(0.9%) | 0.075 |
| Mental disturbance, n (%) | 789(0.3%) | 58(0.4%) | 0.020 |
| Previous myocardial infarction, n (%) | 4736(1.6%) | 369(2.4%) | 0.059 |
| Liver or kidney insufficiency, n (%) | 2521(0.8%) | 243(1.5%) | 0.067 |
| Dyslipidemia, n (%) | 23062(7.9%) | 2325(15.9%) | **0.248** |
| PVD, n (%) | 4498(1.5%) | 652(4.2%) | **0.165** |
| COPD, n (%) | 3091(1.0%) | 228(1.4%) | 0.040 |
| Lung infection within 2 weeks prior to admission, n (%) | 994(0.3%) | 111(0.7%) | 0.053 |
| Urinary tract infection within 2 weeks prior to admission, n (%) | 581(0.2%) | 50(0.3%) | 0.025 |
| **Medications prior to admission** |  |  |  |
| Antiplatelet, n (%) | 59281(19.9%) | 7238(47.8%) | **0.616** |
| Anticoagulation, n (%) | 8788(2.9%) | 945(6.2%) | **0.157** |
| Antihypertensive, n (%) | 145038(48.8%) | 9568(62.3%) | **0.275** |
| Diabetic medication, n (%) | 52433(17.4%) | 3761(24.3%) | **0.170** |
| Chinese patent drug, n (%) | 24088(8.2%) | 2933(19.8%) | **0.338** |
| **Arrival and admission information** |  |  |  |
| Time from onset to door(hour), median [IQR] | 20[4.1;48] | 15.8[3;48] | 0.030 |
| NIHSS on presentation |  |  | **0.154** |
| 0, n (%) | 40198(13.1%) | 1685(10.7%) |  |
| 1, n (%) | 56555(18.5%) | 2401(15.3%) |  |
| 2, n (%) | 76485(25.0%) | 3736(23.7%) |  |
| 3, n (%) | 55943(18.3%) | 3070(19.5%) |  |
| 4, n (%) | 47014(15.3%) | 2870(18.2%) |  |
| 5, n (%) | 30208(9.9%) | 1970(12.5%) |  |
| Hospital level |  |  | 0.087 |
| 2, n (%) | 115125(37.6%) | 6578(41.8%) |  |
| 3, n (%) | 191278(62.4%) | 9154(58.2%) |  |
| Where patient first received care |  |  | 0.057 |
| Neurology emergency, n (%) | 65538(21.9%) | 3328(21.8%) |  |
| Medical emergency, n (%) | 100237(33.5%) | 4763(31.2%) |  |
| Neurosurgery emergency, n (%) | 942(0.3%) | 45(0.3%) |  |
| Outpatient clinic, n (%) | 132078(44.2%) | 7146(46.8%) |  |
| **Physical and laboratory examination** |  |  |  |
| Height(cm), median [IQR] | 167[160;172] | 168[160;172] | 0.033 |
| Weight(kg), median [IQR] | 65[60;72] | 65[60;74] | 0.049 |
| Pulse, median [IQR] | 76[69;82] | 76[68;82] | 0.029 |
| Systolic blood pressure(mmHg), median [IQR] | 149[134;163] | 149[134;162] | 0.018 |
| Diastolic blood pressure(mmHg), median [IQR] | 86[80;95] | 85[78;95] | 0.035 |
| Platelet(10^9^/L), median [IQR] | 205[165;248] | 208[167;250] | 0.036 |
| INR, median [IQR] | 1[0.93;1.06] | 1[0.94;1.08] | 0.089 |
| Low-density lipoprotein(mmol/L), median [IQR] | 2.7[2.11;3.32] | 2.57[1.97;3.22] | 0.017 |
| Fasting glucose(mmol/L), median [IQR] | 5.55[4.91;6.77] | 5.64[4.97;7.1] | 0.054 |
| Hemoglobin A1C(%), median [IQR] | 5.8[5.2;6.6] | 5.845[5.3;6.8925] | 0.060 |
| BUN(mmol/L) , median [IQR] | 5.2[4.2;6.45] | 5.27[4.26;6.55] | 0.045 |
| Uric acid(μmol/L), median [IQR] | 306[244.1;372.8] | 304[244;370.8] | 0.002 |
| Serum creatinine(μmol/L), median [IQR] | 70[58.2;85] | 70.3[58.7;85.4] | 0.032 |
| HCY(μmol/L), median [IQR] | 13.7[10.2;19.1] | 14.5[10.9;20] | 0.073 |
| **In-hospital treatment and complications** |  |  |  |
| Antiplatelet, n (%) | 268922(89.2%) | 13148(88.0%) | 0.040 |
| IV Urokinase thrombolysis, n (%) | 4998(1.6%) | 283(1.8%) | 0.013 |
| IV t-PA thrombolytic, n (%) | 16010(5.3%) | 1000(6.4%) | 0.049 |
| Dysphagia, n (%) | 11646(4.4%) | 1197(8.8%) | **0.178** |

Abbreviations: SMD, standardized mean differences; IQR, interquartile range; TIA, transient ischemic attack; ICH, intra-cerebral hemorrhage; SAH, subarachnoid hemorrhage; PVD, peripheral vascular disease; COPD, chronic obstructive pulmonary disease; NIHSS, national institutes of health stroke scale; INR, international normalized ratio; BUN, blood urea nitrogen; HCY, homocysteine; IV, intravenous; t-PA, tissue plasminogen activator.

**Table S2** Comparative analysis of baseline characteristics pre- and post-2:1 negative-to-positive class downsampling in patients with complete candidate variables.

| **Characteristics** | **Pre-**  **downsampling**  **(n=185,774)** | **Post-**  **downsampling**  **(n=25,608)** | **SMD** |
| --- | --- | --- | --- |
|  |  |  |  |
| **Demographics** |  |  |  |
| Age, median [IQR] | 65[57;74] | 66[58;74] | 0.047 |
| Sex, male, n (%) | 119658(64.4%) | 16656(65.0%) | 0.013 |
| Area |  |  | 0.083 |
| Eastern, n (%) | 50363(27.1%) | 6559(25.6%) |  |
| Southern, n (%) | 21342(11.5%) | 2993(11.7%) |  |
| Northern, n (%) | 36568(19.7%) | 5435(21.2%) |  |
| Central, n (%) | 38680(20.8%) | 5779(22.6%) |  |
| Northeast, n (%) | 15415(8.3%) | 1901(7.4%) |  |
| Southwest, n (%) | 17473(9.4%) | 2049(8.0%) |  |
| Northwest, n (%) | 5933(3.2%) | 892(3.5%) |  |
| Insurance |  |  | 0.038 |
| Urban Employee, n (%) | 57607(31.0%) | 8281(32.3%) |  |
| Urban Resident, n (%) | 36368(19.6%) | 4996(19.5%) |  |
| Rural Resident, n (%) | 74499(40.1%) | 10153(39.6%) |  |
| Self-pay, n (%) | 11371(6.1%) | 1403(5.5%) |  |
| Others, n (%) | 5929(3.2%) | 775(3.0%) |  |
| **Medical History** |  |  |  |
| Smoking |  |  | 0.041 |
| No, n (%) | 111954(60.3%) | 15402(60.1%) |  |
| Quit, n (%) | 24258(13.1%) | 3671(14.3%) |  |
| Yes, n (%) | 49562(26.7%) | 6535(25.5%) |  |
| Drinking |  |  | 0.004 |
| Yes, n (%) | 46354(25.0%) | 6345(24.8%) |  |
| No, n (%) | 139420(75.0%) | 19263(75.2%) |  |
| Previous ischemic stroke, n (%) | 50637(27.3%) | 10650(41.6%) | **0.305** |
| Previous TIA, n (%) | 2163(1.2%) | 385(1.5%) | 0.030 |
| Previous SAH, n (%) | 481(0.3%) | 60(0.2%) | 0.005 |
| Previous ICH, n (%) | 3887(2.1%) | 665(2.6%) | 0.033 |
| Hypertension, n (%) | 119381(64.3%) | 17323(67.6%) | 0.071 |
| Diabetes mellitus, n (%) | 41075(22.1%) | 6178(24.1%) | 0.048 |
| Carotid artery stenosis, n (%) | 2407(1.3%) | 551(2.2%) | 0.066 |
| Atrial fibrillation, n (%) | 6100(3.3%) | 939(3.7%) | 0.021 |
| Heart failure, n (%) | 1248(0.7%) | 250(1.0%) | 0.034 |
| Prosthetic Heart Valve, n (%) | 212(0.1%) | 34(0.1%) | 0.005 |
| Sickle cell, n (%) | 4(0.0%) | 0(0.0%) | 0.007 |
| Current pregnancy or 6 week postpartum, n (%) | 13(0.0%) | 0(0.0%) | 0.012 |
| Dementia, n (%) | 614(0.3%) | 144(0.6%) | 0.035 |
| Mental disturbance, n (%) | 508(0.3%) | 70(0.3%) | <0.001 |
| Previous myocardial infarction, n (%) | 2773(1.5%) | 436(1.7%) | 0.017 |
| Liver or kidney insufficiency, n (%) | 1503(0.8%) | 256(1.0%) | 0.020 |
| Dyslipidemia, n (%) | 14658(7.9%) | 2609(10.2%) | 0.080 |
| PVD, n (%) | 2802(1.5%) | 580(2.3%) | 0.056 |
| COPD, n (%) | 1875(1.0%) | 264(1.0%) | 0.002 |
| Lung infection within 2 weeks prior to admission, n (%) | 624(0.3%) | 115(0.4%) | 0.018 |
| Urinary tract infection within 2 weeks prior to admission, n (%) | 302(0.2%) | 43(0.2%) | 0.001 |
| **Medications prior to admission** |  |  |  |
| Antiplatelet, n (%) | 37329(20.1%) | 7352(28.7%) | **0.202** |
| Anticoagulation, n (%) | 5036(2.7%) | 881(3.4%) | 0.042 |
| Antihypertensive, n (%) | 91023(49.0%) | 13626(53.2%) | 0.084 |
| Diabetic medication, n (%) | 33327(17.9%) | 5087(19.9%) | 0.049 |
| Chinese patent drug, n (%) | 15237(8.2%) | 2845(11.1%) | 0.099 |
| **Arrival and admission information** |  |  |  |
| Time from onset to door(hour), median [IQR] | 20[4.2;48] | 18.9[4;48] | 0.022 |
| NIHSS on presentation |  |  | 0.038 |
| 0, n (%) | 23921(12.9%) | 3121(12.2%) |  |
| 1, n (%) | 34086(18.3%) | 4460(17.4%) |  |
| 2, n (%) | 46678(25.1%) | 6429(25.1%) |  |
| 3, n (%) | 34043(18.3%) | 4768(18.6%) |  |
| 4, n (%) | 28896(15.6%) | 4214(16.5%) |  |
| 5, n (%) | 18150(9.8%) | 2616(10.2%) |  |
| Hospital level |  |  | 0.030 |
| 2, n (%) | 72619(39.1%) | 10388(40.6%) |  |
| 3, n (%) | 113155(60.9%) | 15220(59.4%) |  |
| Where patient first received care |  |  | 0.024 |
| Neurology emergency, n (%) | 39227(21.1%) | 5367(21.0%) |  |
| Medical emergency, n (%) | 64066(34.5%) | 8588(33.5%) |  |
| Neurosurgery emergency, n (%) | 563(0.3%) | 77(0.3%) |  |
| Outpatient clinic, n (%) | 81918(44.1%) | 11576(45.2%) |  |
| **Physical and laboratory examination** |  |  |  |
| Height(cm), median [IQR] | 167[160;172] | 167[160;172] | 0.015 |
| Weight(kg), median [IQR] | 65[60;72] | 65[60;73] | 0.023 |
| Pulse, median [IQR] | 76[68;82] | 76[68;82] | 0.009 |
| Systolic blood pressure(mmHg), median [IQR] | 149[135;163] | 149[135;163] | 0.010 |
| Diastolic blood pressure(mmHg), median [IQR] | 86[79;95] | 86[79;95] | 0.016 |
| Platelet(109/L), median [IQR] | 206[166;248] | 206[167;249] | 0.012 |
| INR, median [IQR] | 1[0.93;1.06] | 1[0.93;1.06] | 0.033 |
| Low-density lipoprotein(mmol/L), median [IQR] | 2.69[2.12;3.31] | 2.66[2.08;3.29] | 0.029 |
| Fasting glucose(mmol/L), median [IQR] | 5.57[4.92;6.8] | 5.6[4.94;6.82] | 0.008 |
| Hemoglobin A1C(%), median [IQR] | 5.7[5.2;6.5] | 5.8[5.2;6.6] | 0.010 |
| BUN(mmol/L) , median [IQR] | 5.2[4.2;6.41] | 5.2 [4.22;6.43] | 0.009 |
| Uric acid(μmol/L), median [IQR] | 306[245;372] | 305[245;371] | 0.002 |
| Serum creatinine(μmol/L), median [IQR] | 70[58;84] | 70[58;84] | 0.005 |
| HCY(μmol/L), median [IQR] | 13.8[10.4;19.03] | 14[10.61;19.39] | 0.023 |
| **In-hospital treatment and complications** |  |  |  |
| Antiplatelet, n (%) | 169975(91.5%) | 23323(91.1%) | 0.015 |
| IV Urokinase thrombolysis, n (%) | 3085(1.7%) | 450(1.8%) | 0.007 |
| IV t-PA thrombolytic, n (%) | 10026(5.4%) | 1366(5.3%) | 0.003 |
| Dysphagia, n (%) | 8055(4.3%) | 1432(5.6%) | 0.058 |

Abbreviations: SMD, standardized mean differences; IQR, interquartile range; TIA, transient ischemic attack; ICH, intra-cerebral hemorrhage; SAH, subarachnoid hemorrhage; PVD, peripheral vascular disease; COPD, chronic obstructive pulmonary disease; NIHSS, national institutes of health stroke scale; INR, international normalized ratio; BUN, blood urea nitrogen; HCY, homocysteine; IV, intravenous; t-PA, tissue plasminogen activator.

## Supplemental Figures


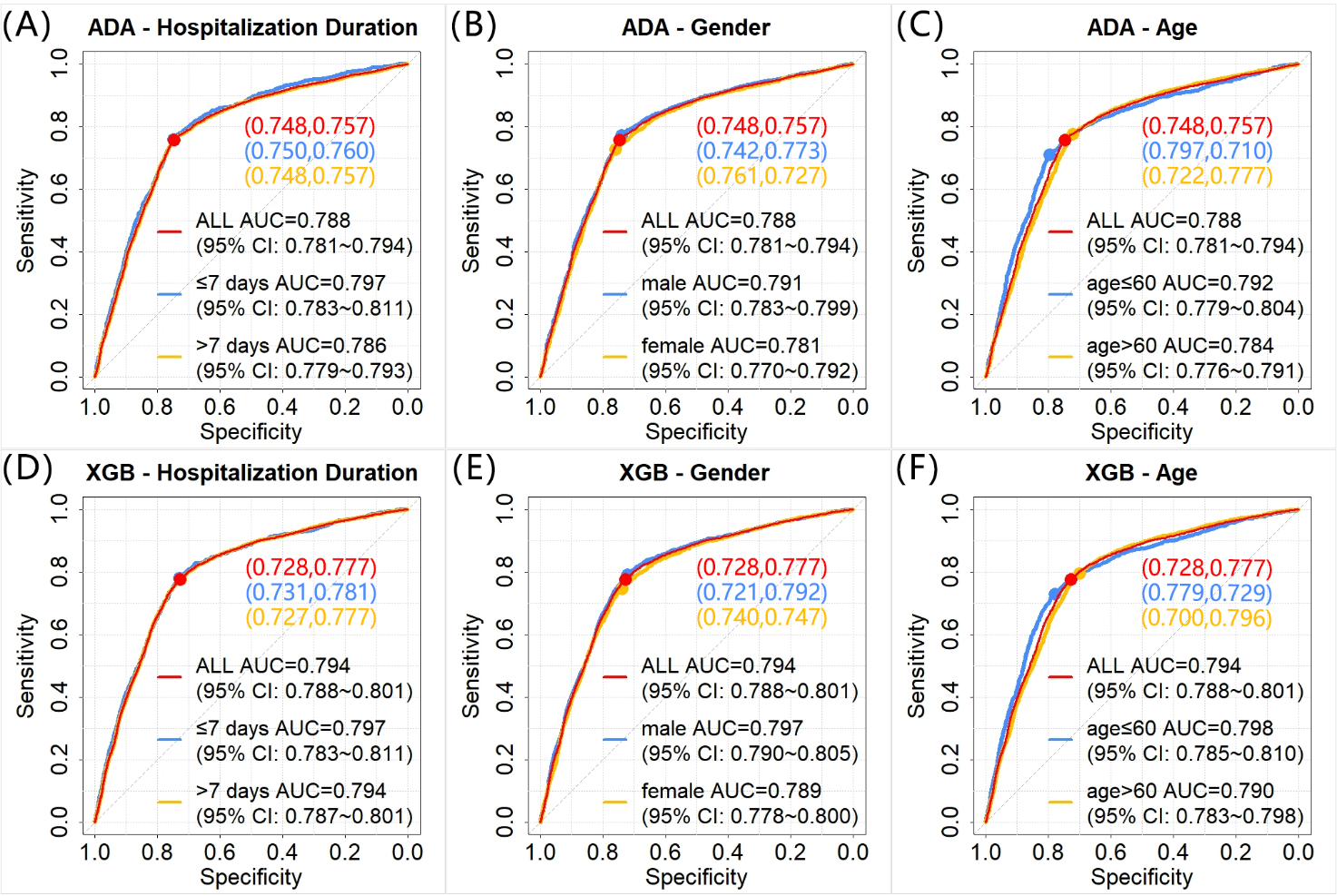
Figure S1. Model performance of the ADA (A, B and C) and XGB (D, E and F) models after combinatorial optimization in the test set, on subgroups based on hospitalization duration (A and D), gender (B and E), and age (C and F). Legend: (Specificity, Sensitivity). Abbreviations: XGB, extreme gradient boosting; ADA, adaptive boosting; AUC, area under ROC curve; ROC, receiver operation characteristic; CI, confidence interval.


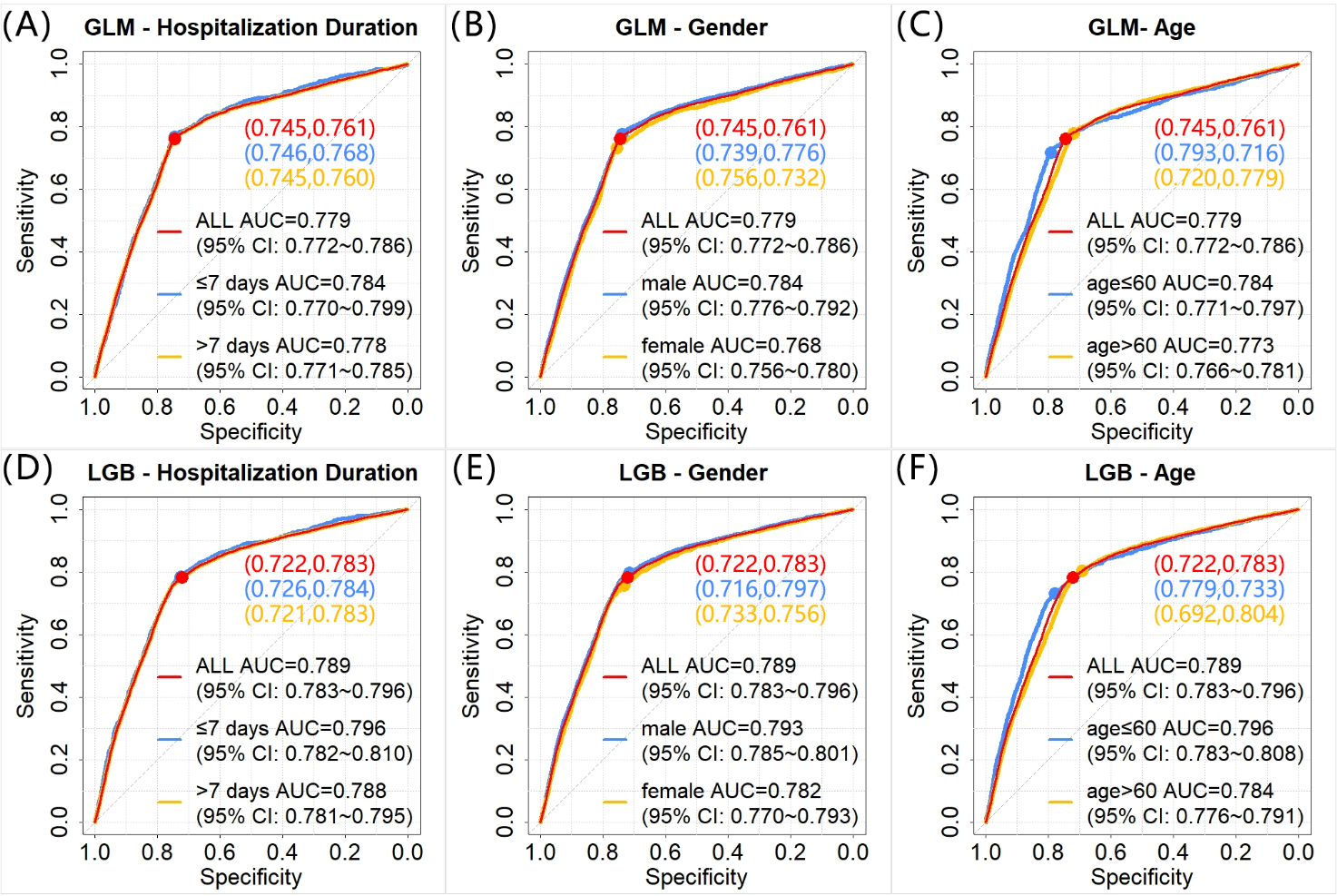
Figure S2. Model performance of the GLM (A, B and C) and LGB (D, E and F) models before combinatorial optimization in the test set, on subgroups based on hospitalization duration (A and D), gender (B and E), and age (C and F). Legend: (Specificity, Sensitivity). Abbreviations: GLM, generalized linear model; LGB, light gradient boosting; AUC, area under ROC curve; ROC, receiver operation characteristic; CI, confidence interval.


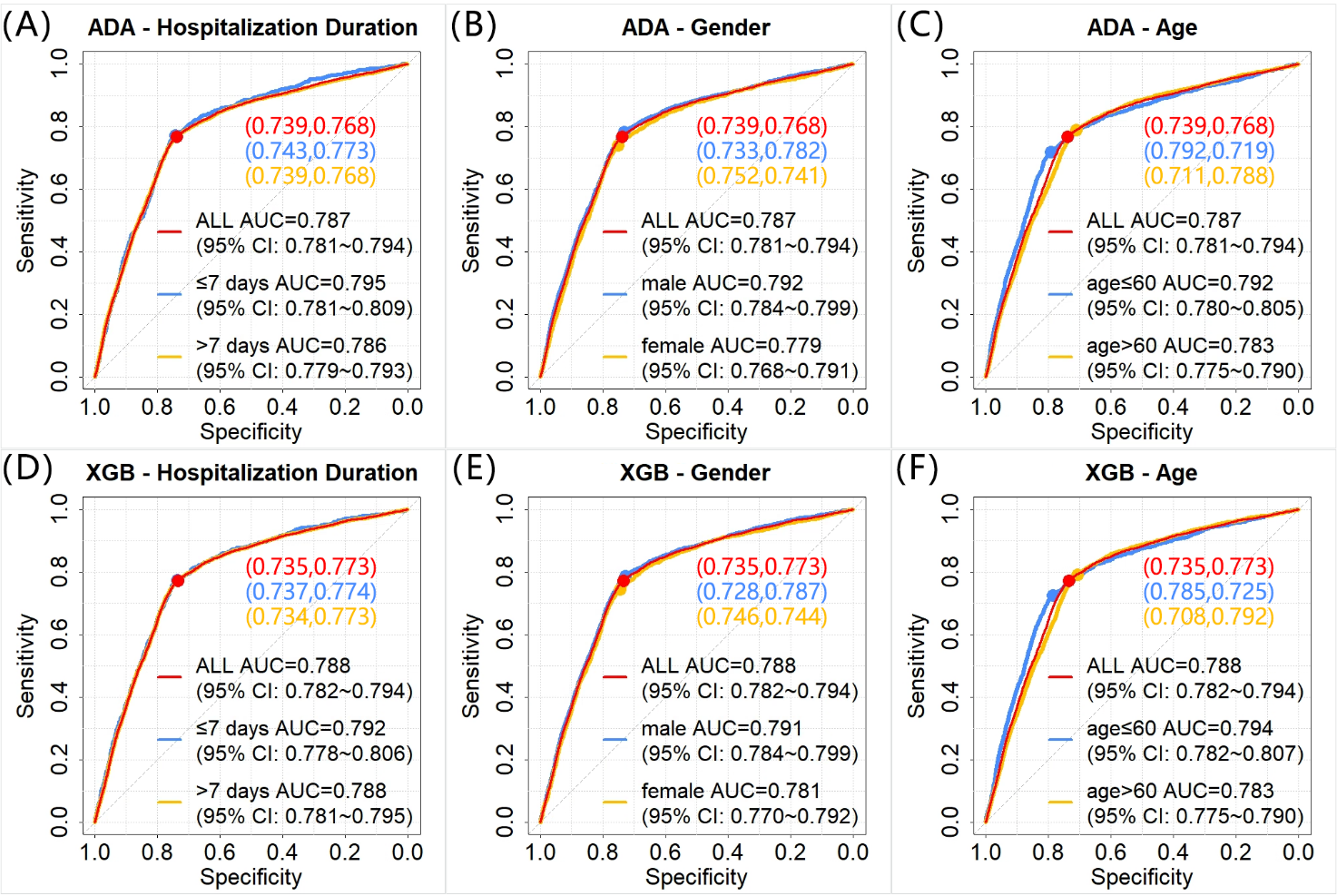
Figure S3. Model performance of the ADA (A, B and C) and XGB (D, E and F) models before combinatorial optimization in the test set, on subgroups based on hospitalization duration (A and D), gender (B and E), and age (C and F). Legend: (Specificity, Sensitivity). Abbreviations: XGB, extreme gradient boosting; ADA, adaptive boosting; AUC, area under ROC curve; ROC, receiver operation characteristic; CI, confidence interval.


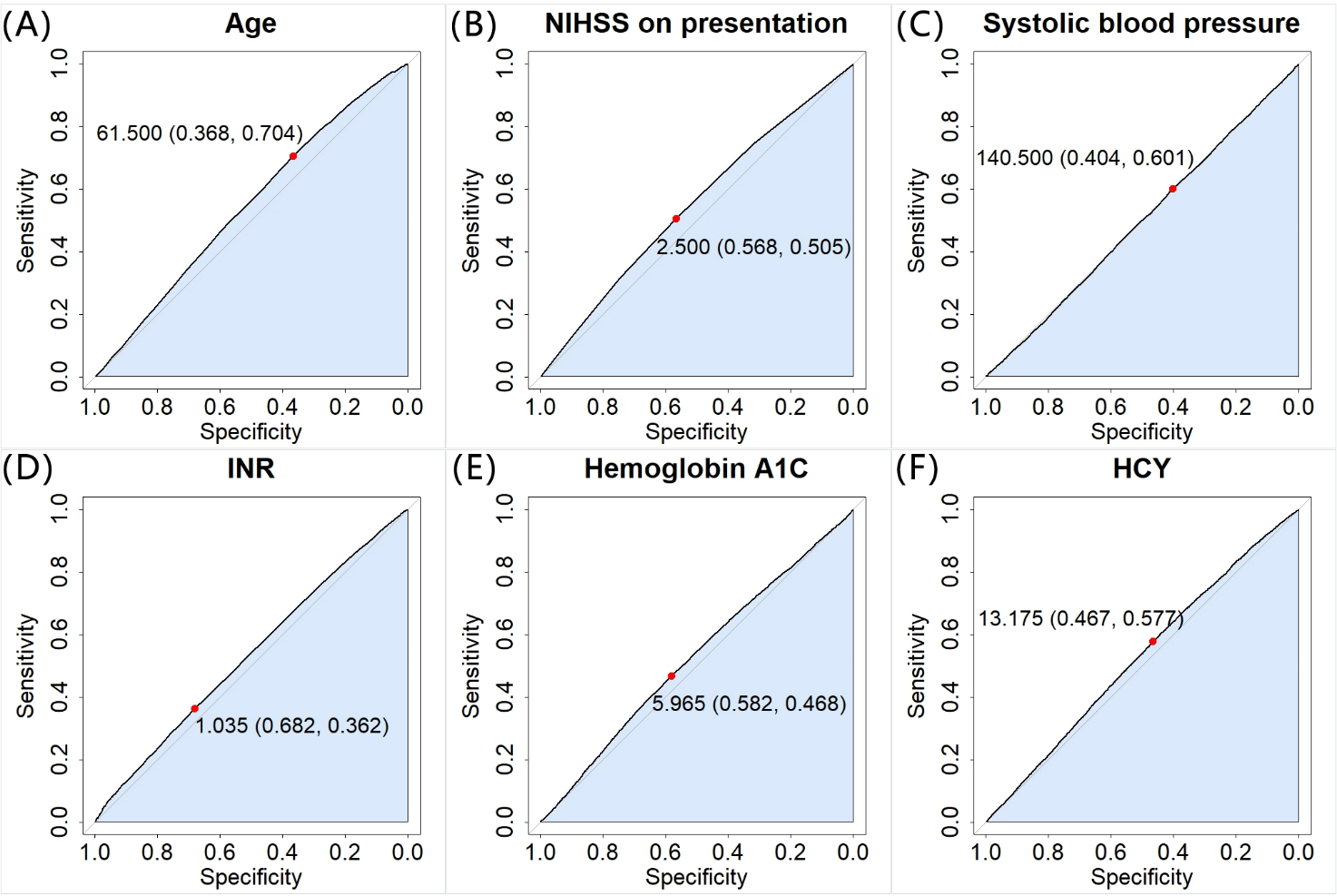
Figure S4. The optimal thresholds of ROC curves for age (A), NIHSS on presentation (B), systolic blood pressure (C), and laboratory examinations including INR (D), Hemoglobin A1C (E), and HCY (F), in predicting in-hospital stroke recurrence. Legend: Optimal threshold (Specificity, Sensitivity). Abbreviations: NIHSS, national institutes of health stroke scale; INR, international normalized ratio; HCY, homocysteine.

## Supplemental Discussion

### 1. Machine learning-selected features underperform logistic regression for predictive modeling

As an alternative strategy, we implemented the Boruta algorithm (a random forest-based feature selection method) to identify predictive variables. This approach selected 14 variables, ranked by descending importance: fasting glucose, blood urea nitrogen, time from onset to door, international normalized ratio, age, NIHSS on presentation, previous ischemic stroke, area, where patient first received care, hospital level, platelet, diabetes mellitus, previous intra-cerebral hemorrhage and liver or kidney insufficiency. Of these, five variables overlapped with those identified through multivariable logistic regression (MLR), though with divergent importance rankings: international normalized ratio, age, NIHSS on presentation, previous ischemic stroke and previous intra-cerebral hemorrhage. Among the nine Boruta-specific variables, three demonstrated limited clinical interpretability, specifically: area, where patient first received care and hospital level.

Using the Boruta-selected features, we reconstructed each prediction model with methodology described in the Method section and evaluated their performance on the test set. The performance metrics are presented in Table S3. The findings indicated that the models built with the variables selected by machine learning-based method systematically underperformed than those constructed using the MLR-selected variables. This suggests that the MLR-selected variables, which offer reasonable interpretability, provide stable and relatively strong predictive performance across various machine learning models.

**Table S3** AUCs and brier score of machine learning models built with different variables.

| Metrics | Feature Selection Method | Individual-mode | | | |
| --- | --- | --- | --- | --- | --- |
|  |  | GLM | XGB | LGB | ADA |
| AUC | MLR | 0.779 | 0.788 | 0.789 | 0.787 |
|  | Random Forest | 0.770 | 0.784 | 0.785 | 0.784 |
|  | p-value | 5e-10 | 0.025 | 0.007 | 0.013 |
| Brier Score | MLR | 0.04335 | 0.04322 | 0.04318 | 0.04319 |
|  | Random Forest | 0.04338 | 0.04325 | 0.04333 | 0.04338 |

Abbreviations: GLM, generalized linear model; XGB, extreme gradient boosting; LGB, light gradient boosting; ADA, adaptive boosting; AUC, area under ROC curve; ROC, receiver operation characteristic; MLR, Multivariable Logistic Regression.

### 2. Comparative analysis with previously reported prediction models

Many stroke recurrence prediction tools have already been published. These risk identification models vary in terms of the populations they target, the predictor variables they use, and the outcome variables they focus on. With reference to the review by Gu^15^, we compare these reported traditional identification models with the model proposed in this study, as shown in the Table S4. The key differences are as follows: First, the populations differ. This study was limited to patients with minor acute ischemic stroke, excluding those with TIA. Second, the outcomes differ. This study focused on the risk of stroke recurrence during hospitalization (excluding TIA recurrence), representing a short-term recurrence risk. In contrast, traditional risk identification models typically evaluate stroke recurrence risk over a fixed period, often medium- to long-term (e.g. 3 months, 6 months, 1 year, 2 years, 5 years, or 10 years). Only models focused on TIA patients assess short-term recurrence risk, usually within a few days, but which includes both TIA and stroke recurrence.

To highlight the differences between the results of this study and the published identification models, we carefully compared the predictor variables used in each method. However, due to missing data for certain predictor variables in the study population, a direct head-to-head comparison with each traditional identification model could not be performed. The specific reasons for this are outlined in Table S5.

After reviewing Table S5, we selected three traditional identification models: ESRS, SPI-I and SPI-II. The availability of their variables and the public accessibility of their scoring rules make them feasible for comparison with the model proposed in this study. Table S6 lists the scoring criteria and risk stratification methods for these four models.

In the test set of this study, we evaluated the predictive ability of three traditional identification models for in-hospital stroke recurrence and compared them with the method developed in this study. Figure S5 displays the ROC curves for ESRS, SPI-I, SPI-II, and the models we developed in this study (GLM, LGB, and RMS) in predicting in-hospital recurrence, along with the corresponding AUC values. The sensitivity, specificity, and Youden’s index at the optimal threshold are listed in Table S7. Moreover, we also examined the sensitivity and specificity of each method for predicting stroke recurrence at other thresholds near the optimal threshold, with the results summarized in Table S8.

The AUC and Youden's index of the SPI-I model are the lowest among the compared methods. At the optimal threshold (predicting stroke recurrence for scores >= 5), the sensitivity is close to 70%, but the specificity is only 40%, leading to a high rate of false positives. Adjusting the threshold to a higher value (predicting stroke recurrence for scores >= 6) improves specificity to 77.6%, but significantly reduces sensitivity to 29.3%. The SPI-I model fails to achieve a good balance between sensitivity and specificity.

Compared to the SPI-I model, the SPI-II model achieves a slightly better balance between sensitivity and specificity. At the optimal threshold (predicting stroke recurrence for scores >= 5), sensitivity exceeds 80%, but specificity remains relatively low at approximately 55%. Adjusting the threshold can improve specificity to 76%, but this results in a significant drop in sensitivity to around 55%. Among the three traditional models, SPI-II performs the best, but it still falls short in effectively predicting in-hospital stroke recurrence.

The ESRS model is similar to the SPI-II model in terms of specificity at the optimal threshold, but its sensitivity is considerably lower, reaching only 70%. As a result, its predictive performance is inferior to that of the SPI-II model.

Compared with the three traditional models, the machine learning models in this study (both GLM and LGB) demonstrate superior predictive performance for in-hospital stroke recurrence. At the optimal threshold, sensitivity exceeds 76% and specificity exceeds 74%, achieving a good balance. From the ROC curves, increasing sensitivity to 80% causes only a slight reduction in specificity to around 70%. Conversely, raising specificity to 80% results in sensitivity ranging from 62% (GLM) to 67% (LGB). Overall, the LGB model emerges as the best predictor among these methods.

Examining the scoring method developed in this study (RMS), at the optimal threshold (predicting stroke recurrence for scores >= 6), its sensitivity and specificity are comparable to the best-performing model (LGB), with sensitivity only 3% lower. Adjusting the threshold to achieve 80% sensitivity or specificity results in the other metric remaining around 65%. Given that scoring methods are more practical and accessible in clinical practice than machine learning models, the RMS scoring system can replace the LGB model as the most effective approach for predicting in-hospital stroke recurrence.

Based on the comparison results, the findings of this study demonstrate superior predictive performance compared to traditional methods. However, we must be very cautious in interpreting this apparent superiority, as the observed difference is likely due to the variations in both the population and outcomes considered by the traditional methods compared to those in this study. The ESRS model applies to an acute ischemic stroke population (not limited to minor strokes), while SPI-I and SPI-II are designed for a mixed population of carotid TIA and minor stroke. The ESRS predicts stroke recurrence within one year, and SPI-I and SPI-II predict recurrence within two years. In contrast, our model is intended for patients with minor ischemic stroke (excluding TIA patients), with the outcome focused on in-hospital stroke recurrence (excluding TIA recurrence). Therefore, a fair comparison between these models is not feasible.

A relatively clear conclusion is that this study is the first to create a model specifically designed to predict in-hospital stroke recurrence in patients with acute minor ischemic stroke, achieving comparatively promising predictive performance. The AUC for predicting in-hospital stroke recurrence is 0.803 (95% CI 0.797–0.809) with the optimal machine learning model and 0.773 (95% CI 0.767–0.780) with a simplified, clinically practical scoring tool (RMS score). As a reference, the AUC for the ESRS in predicting one-year stroke recurrence is 0.59 (95% CI 0.56–0.62)^61^, while SPI-I and SPI-II have AUCs of 0.59 (95% CI, 0.57–0.60) and 0.63 (95% CI, 0.62–0.65)^26^, respectively, for predicting two-year stroke recurrence.

**Table S4** A summary of previously reported stroke recurrence risk prediction models.

| **Prediction**  **Model** | **Year of**  **Publication** | **Target**  **Population** | **Predictive**  **Variables** | **Predictive Outcome: stroke recurrence in** |
| --- | --- | --- | --- | --- |
| FSRJ | 1991 | AIS | 9: age, hypertension, diabetes, smoker, atrial fibrillation, cardiac diseases, chronic kidney disease, non-lacunar stroke, previous ischemic stroke.^21^ | 1 year |
| ESRS | 2005 | AIS | 8: age, hypertension, diabetes, smoker, myocardial infarction, peripheral arterial disease, cardiac diseases, previous ischemic stroke.^22,23^ | 1 year |
| RRE-90-A | 2010 | AIS | 2: history of stroke or TIA within 1 month prior to stroke onset, stroke subtype based on the Causative Classification of Stroke System.^20,24^ | 90 days |
| RRE-90-B | 2010 | AIS | 6: history of stroke or TIA within 1 month prior to stroke onset, stroke subtype based on the Causative Classification of Stroke System, presence of multiple infarcts of different ages, simultaneous infarcts in different circulations, multiple acute infarcts, isolated cortical infarcts.^20,24^ | 90 days |
| Hankey | 1992 | TIA | 8: age, gender, peripheral vascular disease, carotid and vertebrobasilar artery TIA, number of TIAs in the past 3 months, transient blurred vision, residual neurological symptoms, left ventricular hypertrophy.^62^ | 5 years |
| California | 2000 | TIA | 5: age, diabetes, duration of symptoms, weakness, language dysfunction.^37^ | 90 days |
| ABCD | 2005 | TIA | 4: age, blood pressure, clinical symptoms, duration of symptoms.^28^ | 7 days |
| ABCD^2^ | 2007 | TIA | 5: age, blood pressure, clinical symptoms, duration of symptoms, diabetes.^38^ | 2, 7, 90 days |
| ABCD^2^-MRI | 2008 | TIA,  minor stroke | 7: age, blood pressure, clinical symptoms, duration of symptoms, diabetes, intracranial arterial stenosis, DWI-positivity.^27^ | 90 days |
| ABCD^2^-I | 2010 | TIA | 6: age, blood pressure, clinical symptoms, duration of symptoms, diabetes, imaging evidence of infarction.^39^ | 7, 90 days |
| ABCD^3^ | 2010 | TIA | 7: age, blood pressure, clinical symptoms, duration of symptoms, diabetes, dual TIAs within 7 days, imaging evidence of infarction.^40^ | 2, 7, 28, 90 days |
| ABCD^3^-I | 2010 | TIA | 8: age, blood pressure, clinical symptoms, duration of symptoms, diabetes, dual TIAs within 7 days, intracranial arterial stenosis, DWI-positivity.^40^ | 2, 7, 28, 90 days |
| ABCDE+ | 2012 | TIA | 6: age, blood pressure, clinical symptoms, duration of symptoms, etiology, DWI-positivity.^41^ | 90 days |
| SPI-I | 1991 | carotid TIA,  minor stroke | 5: age, diabetes, severe hypertension, cardiac diseases, stroke for the index event.^25^ | 2 years |
| SPI-II | 2000 | carotid TIA,  minor stroke | 7: age, diabetes, severe hypertension, coronary artery disease, stroke for the index event, congestive heart failure, prior stroke.^26^ | 2 years |
| Dutch TIA | 1993 | TIA,  minor stroke | 13: age, gender, dysarthria, multiple TIAs, persisting duration, diabetes, intermittent claudication, hematocrit, border zone infarct, any other infarct, white matter hypodensity, anteroseptal infarct, increased terminal P wave.^29^ | 2 years |
| LiLAC | 2005 | TIA,  minor stroke | 10: age, gender, intermittent claudication, diabetes, hypertension, minor stroke or TIA, paresis, dysarthria, white matter lesions, ST-depression.^30^ | 10 years |
| This Study | / | minor acute ischemic stroke (excluding TIA) | 13: age, previous ischemic stroke, previous intra-cerebral hemorrhage, carotid artery stenosis, heart failure, peripheral vascular disease, antiplatelet, NIHSS on presentation, systolic blood pressure, international normalized ratio, hemoglobin A1C, homocysteine, dysphagia. | stroke recurrence  during hospitalization (excluding TIA recurrence) |

**Table S5** The specific reasons why comparisons could not be performed.

| **Prediction**  **Model** | **The reason why a head-to-head comparison with the model proposed in this study could not be performed.** |
| --- | --- |
| FSRJ | Two Variables are not available in the CSCA database used in this study: chronic kidney disease, non-lacunar stroke. |
| ESRS | - |
| RRE-90-A | All Variables are not available in the CSCA database used in this study. |
| RRE-90-B | All Variables are not available in the CSCA database used in this study. |
| Hankey | Five Variables are not available in the CSCA database used in this study: carotid and vertebrobasilar artery TIA, number of TIAs in the past 3 months, transient blurred vision, residual neurological symptoms, left ventricular hypertrophy. |
| California | The two variables-clinical symptoms [unilateral weakness, speech disturbance without weakness, other] and duration of symptoms in min [>=60, 10-59, <10]-used in these models were specifically designed for TIA patients. In the CSCA database utilized in this study, these variables are required only for TIA patients and are not applicable to non-TIA minor stroke patients. Consequently, these variables are missing from the population included in this study. |
| ABCD |  |
| ABCD^2^ |  |
| ABCD^2^-MRI |  |
| ABCD^2^-I |  |
| ABCD^3^ |  |
| ABCD^3^-I |  |
| ABCDE+ |  |
| SPI-I | - |
| SPI-II | - |
| Dutch TIA | 1. Six Variables are not available in the CSCA database used in this study: intermittent claudication, border zone infarct, any other infarct, white matter hypodensity, anteroseptal infarct, increased terminal P wave.  2. The Dutch TIA did not convert the model into a score but only provided the predictor variables and their relative effects. |
| LiLAC | 1. Four Variables are not available in the CSCA database used in this study: intermittent claudication, paresis, white matter lesions, ST-depression.  2. The LiLAC did not convert the model into a score but only provided the predictor variables and their relative effects. |

**Table S6** Scoring criteria and risk stratification of the 4 models to be compared.

| **Prediction Model** | **Scoring Criteria** | **Risk Stratification** |
| --- | --- | --- |
| ESRS | age (65-75 years, 1 point; >75 years, 2 points), hypertension (1 point), diabetes (1 point), smoker (1 point), myocardial infarction (1 point), peripheral arterial disease (1 point), cardiac diseases (1 point), and previous ischemic stroke (1 point). | low- (<3 points) and high-risk groups (≥3 points) |
| SPI-I | age (>65 years, 3 points), diabetes (3 points), severe hypertension (2 points, SBP>180 mmHg or DBP>100 mmHg), coronary artery disease (1 point) and stroke for the index event (stroke, 2 points; TIA, 0 point). | low- (<3 points), medium- (3 to 6 points) and high-risk groups (≥7 points). |
| SPI-II | age (>70 years, 2 points), diabetes (3 points), severe hypertension (1 point, SBP>180 mmHg or DBP>100 mmHg), coronary artery disease (1 point), stroke for the index event (stroke, 2 points; TIA, 0 point), congestive heart failure (3 points) and prior stroke (3 points). | low- (<4 points), medium- (4 to 7 points) and high-risk groups (≥8 points). |
| This Study | Detailed in Table 3. | low- (<6 points) and high-risk groups (≥6 points) |

**Table S7** Key performance metrics (AUC, sensitivity, specificity, and Youden’s index) at the optimal threshold for the four models to be compared.

| **Prediction Model** | | **AUC** | **Sensitivity** | **Specificity** | **Youden's index** |
| --- | --- | --- | --- | --- | --- |
| ESRS | | 0.674 | 70.0% | 57.1% | 0.271 |
| SPI-I | | 0.556 | 68.6% | 39.8% | 0.084 |
| SPI-II | | 0.727 | 83.3% | 55.6% | 0.389 |
| This Study | GLM | 0.781 | 76.4% | 74.3% | 0.507 |
|  | LGB | 0.803 | 76.6% | 74.3% | 0.508 |
|  | RMS Score | 0.773 | 73.7% | 74.4% | 0.481 |

**Table S8** The sensitivity and specificity of each method at other thresholds near the optimal threshold.

| **Prediction Model** | **Threshold for predicting stroke recurrence** | **Sensitivity** | **Specificity** |
| --- | --- | --- | --- |
| SPI-I | >= 3 | 76.9% | 30.2% |
|  | >= 4 | 74.9% | 31.8% |
|  | >= 5 | 68.6% | 39.8% |
|  | >= 6 | 29.3% | 77.6% |
|  | >= 7 | 22.4% | 82.5% |
| SPI-II | >= 3 | 91.3% | 30.9% |
|  | >= 4 | 88.0% | 40.4% |
|  | >= 5 | 83.3% | 55.6% |
|  | >= 6 | 54.9% | 76.0% |
|  | >= 7 | 46.2% | 81.1% |
| ESRS | >= 1 | 98.5% | 6.7% |
|  | >= 2 | 90.7% | 27.8% |
|  | >= 3 | 70.0% | 57.1% |
|  | >= 4 | 39.4% | 81.4% |
|  | >= 5 | 14.9% | 94.3% |
| RMS | >= 4 | 86.6% | 50.3% |
|  | >= 5 | 80.4% | 66.3% |
|  | >= 6 | 73.7% | 74.4% |
|  | >= 7 | 62.3% | 79.9% |
|  | >= 8 | 44.4% | 86.6% |


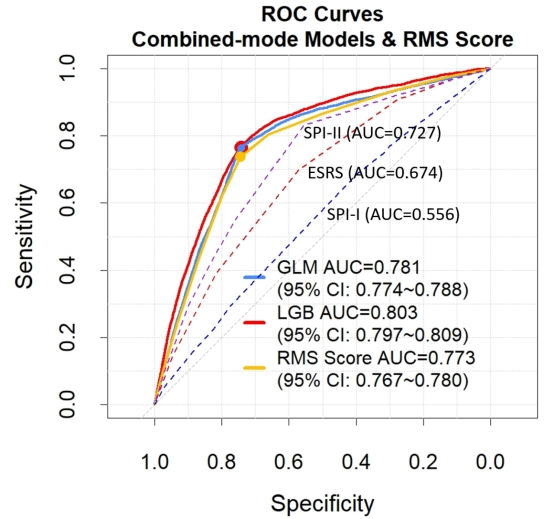


Figure S5 A comparison of the ROC curves for all methods.
